# Supplementary material for: Does a complex intervention targeting communities, health facilities and district health managers increase the utilisation of community-based child health services? A before and after study in intervention and comparison areas of Ethiopia
Source: BMJ Open. 2020 Sep 15;10(9):e040868. doi: 10.1136/bmjopen-2020-040868 (PMC7493123; doi:10.1136/bmjopen-2020-040868)
Supplement: Supplementary data [file bmjopen-2020-040868supp001.pdf]

S1 Table Demographic characteristics, number of different health facilities and staff at baseline (December 2016-February 2017) and endline (December 2018-February 2019) surveys in intervention and comparison districts.

**Characteristic**	**Baseline**		**Endline**	
	Intervention districts n=21[a]	Comparison districts n=25[b]	Intervention districts n=22[c]	Comparison districts n=24 [d]
	Mean (95% CI)	Mean (95% CI)	Mean (95% CI)	Mean (95% CI)
Population size (*1000)	132 (111-152)	139 (121-159)	121 (103-141)	138 (115-162)
Women 15-49 years (*1000)	29 (25-34)	32 (27-38)	28 (22-34)	31 (25-36)
Children <5 years (*1000)	20 (17-23)	21 (18-25)	19 (16-23)	21 (16-26)
Household size	4.8 (4.6-5.0)	4.6 (4.5-4.7)	4.5 (4.2-4.8)	4.6 (4.4-4.9)
Hospitals	0.4 (0.2-0.6)	0.3 (0.1-0.5)	0.3 (0.1-0.5)	0.4 (0.2-0.6)
Health centres	5.3 (4.8-5.8)	5.0 (4.6-5.5)	5.0 (4.4-5.6)	4.8 (4.1-5.5)
Health posts	24 (21-27)	27 (24-30)	22 (19-26)	26 (22-30)
Ambulances	1.6 (1.3-2.0)	1.9 (1.6-2.3)	2.4 (2.1-2.8)	3.1 (2.4-3.8)
Ambulances for child use	1.3 (0.8-1.7)	1.5 (1.0-2.0)	2.0 (1.5-2.5)	2.8 (2.0-3.7)
Health officers per health centre	2.0 (1.7-2.3)	1.9 (1.6-2.3)	2.6 (2.0-3.1)	2.4 (1.9-2.8)
Midwives per health centre	2.5 (2.1-3.0)	2.5 (2.0-3.2)	3.7 (2.6-4.7)	3.1 (2.4-3.8)
Health extension workers per health post	2.3 (2.0-2.7)	2.2 (2.0-2.3)	2.6 (2.3-2.9)	2.8 (1.4-4.2)

[a] Missing data from 5 districts
[b] Missing data from 1 district
[c] Missing data from 4 districts
[d] Missing data from 2 districts

S2 Table Health messages received, meetings attended, and health issues addressed in meetings among households participating at baseline (December 2016- February 2017) and endline surveys (December 2018-February 2019) in intervention and comparison areas

Categories	**Baseline household survey**		**Endline household survey**			
	Intervention N=1240	Comparison N=1264	Intervention N=2434	Comparison N=2247	Difference in Difference[a]	P-value[b]
**Seen or heard messages on**						
Diarrhoea treatment	60 (55-65)	49 (43-55)	52 (49-56)	44 (40-48)	-3	0.03
Pneumonia treatment	29 (24-35)	27 (22-32)	26 (22-30)	22 (18-26)	2	0.76
Sick newborn treatment	39 (33-46)	34[c] (28-40)	31 (26-35)	27 (23-31)	-1	0.87
**Participated in developing local maternal, newborn and child health action plan in the last 12 months organized by[c]:**						
Kebele health team	1 (1-2)	1 (0-1)	1 (0-2)	1 (0-2)	0	0.25
Health extension worker	3 (2-5)	1 (1-2)	1 (1-2)	1 (0-1)	-2	0.04
**Attended meeting to discuss on maternal, newborn and child health issues in the past 12 months organized by[d]:**						
Kebele health team	2 (1-3)	1 (0-2)	1 (1-2)	1 (0-1)	-1	0.08
Health extension worker	3 (2-5)	2 (1-3)	2 (1-2)	1 (0-1)	0	0.31

[a] Difference-in-Difference: the difference in the proportion between intervention and comparison areas at endline subtracted from the difference in proportion between intervention and comparison at baseline.

[b] P-value obtained from a logistic regression model for the Difference-in-Difference analysis

[c] Missing data from one individual

[d] Missing data for one individual in intervention areas and 11 individuals in comparison areas at baseline and for one individual in intervention and comparison areas each at endline.

[e] Missing data for two individuals in intervention areas and 11 individuals in comparison areas at baseline and for one individual in intervention and comparison areas each at endline.

S3 Table Caregivers' unprompted knowledge of newborn danger signs at baseline (December 2016-February 2017) and endline surveys (December 2018-February 2019) in intervention and comparison areas.

	**Baseline household survey**		**Endline household survey**		Difference in Difference[a]	P-value[b]
	Intervention n=1259	Comparison n=1273	Intervention n=2434	Comparison n=2247		
	% (95%CI)	% (95%CI)	% (95%CI)	% (95%CI)	%	
**Signs of newborn illness**						
Reduced feeding	60 (55-65)	59 (53-64)	53 (49-58)	51 (47-56)	1	0.16
Difficult or fast breathing	31 (26-36)	38 (33-43)	37[c] (34-41)	35 (32-38)	9	0.06
Movement only when stimulated or no moment even when stimulated	11 (8-15)	20 (16-24)	18[c] (16-21)	19 (16-23)	8	<0.01
Unusually hot or cold	49 (43-54)	42 (38-48)	64[c] (60-69)	59 (53-64)	-2	0.05
Convulsions	7 (5-9)	9 (6-12)	14[c] (12-17)	12 (10-15)	4	0.35
Severe chest in-drawing	6 (4-8)	4 (2-6)	10[c] (8-12)	7 (5-10)	1	0.33
Yellow palms/soles/eyes	1 (1-2)	2 (1-4)	7[c] (5-9)	6 (3-8)	2	0.30
Diarrhoea	54 (50-59)	52 (47-58)	60[c] (55-64)	53 (49-57)	5	0.59
Skin pustules	5 (3-7)	7 (5-9)	10[c] (8-13)	8 (6-11)	4	0.03
Cord red or draining pus	1 (1-2)	1 (1-2)	6[c] (4-8)	5 (3-7)	1	0.56
Small infant (weigh less than 2000 grams)	1 (1-2)	1 (0-1)	5[c] (3-7)	4 (2-6)	1	0.83

[a] Difference-in-Difference: the difference in the proportion between intervention and comparison areas at endline subtracted from the difference in proportion between intervention and comparison at baseline.
[b] P-value obtained from a logistic regression model for the Difference-in-Difference analysis
[c] Missing data from one individual

S4 Table Caregivers' knowledge on actions to be taken when a child under five years is sick at baseline (December 2016- February 2017) and endline surveys (December 2018-February 2019) in intervention and comparison areas.

	**Baseline household survey**		**Endline household survey**			
	Intervention n=1259	Comparison n=1273	Intervention n=2434	Comparison n=2247	Difference in Difference[a]	P-value[b]
	% (95%CI)	% (95%CI)	% (95%CI)	% (95%CI)	%	
**Action taken when child has:**						
**Fever**						
Go to health extension worker	20 (16-25)	22 (17-27)	26 (22-31)	25 (21-30)	3	0.26
Go to health centre	83 (79-87)	84 (79-87)	88 (85-90)	90 (87-92)	-1	0.77
**Diarrhoea**						
Go to health extension worker	20 (16-25)	21 (17-27)	27 (22-32)	24 (20-29)	4	0.48
Go to health centre	86 (82-89)	86 (81-89)	90 (86-92)	90 (88-92)	0	0.84
**Respiratory infection or cough**						
Go to health extension worker	20 (16-24)	22 (17-27)	26 (21-30)	24 (20-29)	4	0.64
Go to health centre	86 (83-89)	86 (82-90)	87 (83-89)	89 (86-91)	-2	0.62
**Signs of newborn sepsis[c]**						
Go to health extension worker	18 (14-22)	20 (15-26)	23 (19-27)	22 (18-26)	3	0.33
Go to health centre	87 (83-90)	86 (82-90)	87[d] (85-88)	89 (87-91)	-3	0.30

[a] Difference-in-Difference: the difference in the proportion between intervention and comparison areas at endline subtracted from the difference in proportion between intervention and comparison at baseline.

[b] P-value obtained from a logistic regression model for the Difference-in-Difference analysis

[c] Caregivers were read the dangers signs indicating neonatal sepsis: reduced feeding, difficult or fast breathing, movement when stimulated or no movement even when stimulated, unusually hot or cold, convulsion, severe chest in-drawing

[d] Missing data from one individual

S5 Table Health post characteristics in baseline (December 2016- February 2017) and endline surveys (December 2018-February 2019) at intervention and comparison areas.

	**Baseline health post survey**		**Endline health post survey**			
	Intervention n=145	Comparison n=131	Intervention n=141	Comparison n=133	Difference in Difference[a]	P-value[b]
**Characteristics of health posts:**	% (95%CI)	% (95%CI)	% (95%CI)	% (95%CI)	%	
Fully staffed[c]	89 (82-93)	79 (70-85)	89 (82-93)	86 (78-91)	-7	0.55
Open less than five days a week	20 (13-29)	31 (21-42)	10 (6-17)	20 (13-30)	1	0.99
Operational days posted	34 (25-452)	31 (22-42)	23 (16-34)	16 (9-25)	4	0.76
Operational hours posted	27 (18-38)	25 (17-35)	13 (8-22)	13 (7-22)	-2	0.88

[a] Difference in Difference: the difference in the proportion between intervention and comparison areas at endline subtracted from the difference in proportion between intervention and comparison at baseline.
[b] P-value obtained from a logistic regression model for the Difference-in-Difference analysis
[c] Two or more health extension workers per health post

S6 Table Supervision at baseline (December 2016- February 2017) and endline surveys (December 2018-February 2019) in intervention and comparison areas.

	**Baseline frontline worker survey**		**Endline frontline worker survey**			
	Intervention	Comparison	Intervention	Comparison	Difference in Difference[a]	P-value[b]
**Supervision**	% (95%CI) [n]	% (95%CI) [n]	% (95%CI) [n]	% (95%CI) [n]	%	
**Health centre**: supervision received in the last 3 months	66 (55-76) [n=83]	61 (50-70) [n=92]	64 (52-75) [n=70]	62 (50-73) [n=71]	-3	0.72
**Health extension worker supervision:** supervision received in last month	59 (48-68) [n=145]	60 (49-69) [n=131]	57 (47-67) [n=141]	57 (47-67) [n=133]	1	0.90
**Health extension worker:** performance review and clincial mentoring meeting in the last 6 months	46 (36-55) [n=145]	41 (32-51) [n=131]	51 (42-60) [n=141]	41 (31-50) [n=133]	5	0.46
**Women's development army leaders:** met with health extension workers and other women development army leaders in the last 3 months	67 (56-76) [n=93]	60 (49-69) [n=94]	64 (55-72) [n=169]	60 (52-69) [n=167]	-3	0.357

[a] Difference-in-Difference: the difference in the proportion between intervention and comparison areas at endline subtracted from the difference in proportion between intervention and comparison at baseline.
[b] P-value obtained from a logistic regression model for the Difference-in-Difference analysis

Berhanu D, *et al. BMJ Open* 2020; 10:e040868. doi: 10.1136/bmjopen-2020-040868
