# Supplementary material for: Does a complex intervention targeting communities, health facilities and district health managers increase the utilisation of community-based child health services? A before and after study in intervention and comparison areas of Ethiopia
Source: BMJ Open. 2020 Sep 15;10(9):e040868. doi: 10.1136/bmjopen-2020-040868 (PMC7493123; doi:10.1136/bmjopen-2020-040868)
Supplement: Author's manuscript [file bmjopen-2020-040868.draft_revisions.pdf]

BMJ Open

BMJ Open is committed to open peer review. As part of this commitment we make the peer review history of every article we publish publicly available.

When an article is published we post the peer reviewers' comments and the authors' responses online. We also post the versions of the paper that were used during peer review. These are the versions that the peer review comments apply to.

The versions of the paper that follow are the versions that were submitted during the peer review process. They are not the versions of record or the final published versions. They should not be cited or distributed as the published version of this manuscript.

BMJ Open is an open access journal and the full, final, typeset and author-corrected version of record of the manuscript is available on our site with no access controls, subscription charges or pay-per-view fees (http://bmjopen.bmj.com).

If you have any questions on BMJ Open's open peer review process please email info.bmjopen@bmj.com

BMJ Open

Effectiveness of a complex intervention to increase utilization of community-based child health services in Ethiopia

Journal:	*BMJ Open*
Manuscript ID	bmjopen-2020-040868
Article Type:	Original research
Date Submitted by the Author:	27-May-2020
Complete List of Authors:	Berhanu, Della; London School of Hygiene and Tropical Medicine Faculty of Infectious and Tropical Diseases, Department of Disease Control; Ethiopian Public Health Institute,<br>Okwaraji, Yemisrach; London School of Hygiene & Tropical Medicine, Department of Control of Disease<br>Defar, Atkure ; Ethiopian Public Health Institute, Health Systems and Reproductive Health Research Directorate; University of Gondar College of Medicine and Health Sciences<br>Bekele, Abebe; Ethiopian Public Health Institute, Health Systems and Reproductive Health Research Directorate<br>Lemango, Ephrem Tekle; Ethiopia Ministry of Health, Maternal and Child Health Directorate<br>Medhanyie, Araya; Mekelle University College of Health Sciences<br>Wordofa, Muluemebet; Jimma University, Department of Population and Family Hea<br>Yitayal, Mezgebu ; University of Gondar College of Medicine and Health Sciences<br>W/Gebriel, Fitsum ; Hawassa University College of Medicine and Health Sciences<br>Desta, Alem ; Mekelle University College of Health Sciences<br>Gebregizabher , Fisseha; Mekelle University College of Health Sciences<br>Daka, Dawit; Jimma University, Department of Health Economics<br>Hunduma, Alemayehu; Jimma University, Department of Population and Family Health<br>Beyene, Habtamu; Hawassa University College of Medicine and Health Sciences<br>Getahun, Tigist ; University of Gondar College of Medicine and Health Sciences, College of Medicine and Health Science, Institute of Public Health<br>Getachew, Theodros ; Ethiopian Public Health Institute, Health System and Reproductive Health Research Directorate; University of Gondar College of Medicine and Health Sciences<br>Woldemariam, Amare; University of Gondar College of Medicine and Health Sciences, Department of Human Nutrition<br>Wolassa, Desta ; Ethiopian Public Health Institute,<br>Persson, Lars; London School of Hygiene and Tropical Medicine; Ethiopian Public Health Institute<br>Schellenberg, Joanna; London School of Hygiene and Tropical Medicine, Disease Control

Keywords:	Epidemiology < TROPICAL MEDICINE, Community child health < PAEDIATRICS, Epidemiology < INFECTIOUS DISEASES

For peer review only - http://bmjopen.bmj.com/site/about/guidelines.xhtml

I, the Submitting Author has the right to grant and does grant on behalf of all authors of the Work (as defined in the below author licence), an exclusive licence and/or a non-exclusive licence for contributions from authors who are: i) UK Crown employees; ii) where BMJ has agreed a CC-BY licence shall apply, and/or iii) in accordance with the terms applicable for US Federal Government officers or employees acting as part of their official duties; on a worldwide, perpetual, irrevocable, royalty-free basis to BMJ Publishing Group Ltd ("BMJ") its licensees and where the relevant Journal is co-owned by BMJ to the co-owners of the Journal, to publish the Work in this journal and any other BMJ products and to exploit all rights, as set out in our licence.

The Submitting Author accepts and understands that any supply made under these terms is made by BMJ to the Submitting Author unless you are acting as an employee on behalf of your employer or a postgraduate student of an affiliated institution which is paying any applicable article publishing charge ("APC") for Open Access articles. Where the Submitting Author wishes to make the Work available on an Open Access basis (and intends to pay the relevant APC), the terms of reuse of such Open Access shall be governed by a Creative Commons licence – details of these licences and which Creative Commons licence will apply to this Work are set out in our licence referred to above.

Other than as permitted in any relevant BMJ Author's Self Archiving Policies, I confirm this Work has not been accepted for publication elsewhere, is not being considered for publication elsewhere and does not duplicate material already published. I confirm all authors consent to publication of this Work and authorise the granting of this licence.

For peer review only

Effectiveness of a complex intervention to increase utilisation of community-based child health services in Ethiopia.

Della Berhanu[1,2*], Yemisrach B. Okwaraji[1,2], Atkure Defar[2,3], Abebe Bekele Belayneh[2], Ephrem Tekle Lemango[4], Araya Abrha Medhanyie[5], Muluemebet Abera Wordofa[6], Mezgebu Yitayal[7], Fitsum W/Gebriel[8], Alem Desta[5], Fisseha Ashebir[5,9], Dawit Wolde Daka[10], Alemayehu Hunduma[6,11], Habtamu Beyene[12,13], Tigist Getahun[3], Theodros Getachew [2,3], Amare Tariku[14] , Desta Wolassa[2], Lars Åke Persson[1,2], Joanna Armstrong Schellenberg[1]

[1] Department of Disease Control, London School of Hygiene & Tropical Medicine, London, United Kingdom.

[2] Health Systems and Reproductive Health Research Directorate, Ethiopian Public Health Institute, Addis Ababa, Ethiopia

[3] Institute of Public Health, College of Medicine and Health Sciences, University of Gondar, Gondar, Ethiopia

[4] Maternal and Child Health Directorate, Federal Ministry of Health, Addis Ababa, Ethiopia

[5] School of Public Health, College of Health Sciences, Mekelle University, Mekelle, Ethiopia

[6] Department of Population and Family Health, Institute of Family Health, Public Health Faculty, Jimma University, Jimma, Ethiopia.

[7] Department of Health Systems and Policy, Institute of Public Health, College of Medicine and Health Sciences, University of Gondar, Gondar, Ethiopia

[8] College of Medicine and Health Sciences, Hawassa University, Hawassa, Ethiopia

[9] Tigray Regional Health Bureau, Tigray, Ethiopia

[10] Department of Health Economics, Management and Policy Public Health Faculty, Jimma University, Jimma, Ethiopia

[11] Addis Ababa City Administration health Bureau, Addis Ababa, Ethiopia.

[12] College of Medicine and Health Sciences, Hawassa University, Hawassa, Ethiopia

[13] Southern Nations, Nationalities and Peoples Regional Health Bureau, Hawassa, Ethiopia

[14] Department of Human Nutrition, Institute of Public Health, College of Medicine and Health Sciences, University of Gondar, Gondar, Ethiopia

*Corresponding author: Della Berhanu; PhD, della.berhanu@lshtm.ac.uk, London School of Hygiene & Tropical Medicine, Keppel St, London WC1E 7HT

Co-authors: Yemisrach B. Okwaraji; PhD, Yemisrach.Okwaraji@lshtm.ac.uk

For peer review only - http://bmjopen.bmj.com/site/about/guidelines.xhtml

For peer review only

Atkure Defar; MPH, MBA, atkuredefar@gmail.com

Abebe Bekele Belayneh; MSc, abebe1277belay@gmail.com

Ephrem Tekle Lemango; MD, MA, ephremt.lemango@gmail.com

Araya Abrha Medhanyie; PhD, araya.medhanyie@gmail.com

Muluemebet Abera Wordofa; PhD, mulu_abera.ts2009@yahoo.com

Mezgebu Yitayal; PhD, mezgebuy@gmail.com

Fitsum W/Gebriel; MD, fitsedememmj@gmail.com

Alem Desta; MSc, alemdw@gmail.com

Fisseha Ashebir; MPH, fmhn2009@gmail.com

Dawit Wolde Daka; MSc, dave86520@gmail.com

Alemayehu Hunduma; MPH, hhalemayehu25@gmail.com

Habtamu Beyene; MSc, habtamu3346@yahoo.com

Tigist Getahun; MPH, tgforblessing@gmail.com

Theodros Getachew; MSc, tedi.getachew@yahoo.com

Amare Tariku; MPH, amaretariku15@yahoo.com

Desta Wolassa; MSc, desta.wolassa@lshtm.ac.uk

Lars Åke Persson; MD, PhD, Lars.Persson@lshtm.ac.uk

Joanna Armstrong Schellenberg; PhD, Joanna.Schellenberg@lshtm.ac.uk

For peer review only

For peer review only - http://bmjopen.bmj.com/site/about/guidelines.xhtml

ABSTRACT

Introduction Ethiopia successfully reduced mortality in children below five years of age during the past few decades, but the utilization of child health services was still low. Optimizing the Health Extension Program was a two-year intervention in 26 districts, focusing on community engagement, capacity strengthening of primary care workers, and reinforcement of district accountability of child health services. We report the intervention's effectiveness on care utilization for common childhood illnesses.

Methods We included a representative sample of 5,773 households with 2,874 under-five children at baseline (December 2016 to February 2017) and 10,785 households and 5,639 under-five children at endline surveys (December 2018 to February 2019) in intervention and comparison areas. Health facilities were also included. We assessed the effect of the intervention using difference-in-differences analyses.

Results There were 31 intervention activities; many were one-off and implemented late. In eight districts, activities were interrupted for four months. Care-seeking for any illness in the two weeks before the survey for children aged 2-59 months at baseline was 58% (95% CI 47-68) in intervention and 49% (95% CI 39-60) in comparison areas. At end-line it was 39% (95% CI 32-45) in intervention and 34% (95% CI 27-41) in comparison areas (difference-in-differences -4 percentage points, adjusted odds ratio (AOR) 0.49,95%CI 0.12-1.95). The intervention neither had an effect on care-seeking among sick neonates, nor on household participation in community engagement forums, supportive supervision of primary care workers, nor on indicators of district accountability for child health services.

Conclusion We found no evidence to suggest that the intervention increased the utilization of care for sick children. The lack of effect could partly be attributed to the short implementation period of a complex intervention and implementation interruption. Future funding schemes should take into

For peer review only - http://bmjopen.bmj.com/site/about/guidelines.xhtml

consideration that complex interventions that include behavior change may need an extended implementation period.

Trial registration: Current Controlled Trials ISRCTN12040912, http://www.isrctn.com/ISRCTN12040912

For peer review only

For peer review only - http://bmjopen.bmj.com/site/about/guidelines.xhtml

Article summary

Strengths and limitations of the study

- We conducted a rigorous effectiveness evaluation of an intervention to increase the utilization of services for sick children under-5 children using baseline (December 2016 to February 2017) and endline (December 2018 to February 2019) surveys, in intervention and comparison areas located in 56 districts across four regions of Ethiopia.
- Data were triangulated with service utilization records from health posts and health centres, which supported the household-level findings of no change in service utilization as a result of the intervention.
- Although only few of the household characteristics differed between intervention and comparison areas overtime and had only a marginal influence on the analyses, it is possible that unmeasured confounders might contribute to the observed results.
- At baseline, the overall proportion of reported illness in the last two weeks was lower than anticipated, and this was higher at the endline indicating that the difference in the reported proportion of sick children could be due to differences in the interaction and the extent of the probing between data collectors and families when enquiring about childhood illnesses.
- Data collection blinded to the allocation, i.e., whether the district where they collected data was an intervention or comparison area indicating that any differential reporting of care utilization between intervention and comparison areas is, therefore, unlikely.

For peer review only - http://bmjopen.bmj.com/site/about/guidelines.xhtml

For peer review only

INTRODUCTION

In the period 1990-2015, Ethiopia reduced under-five mortality by 67% (1). The Ethiopian Demographic and Health Surveys (DHS) showed infant mortality rates at 77, 48, to 43 deaths per 1000 live births, in 2005, 2016, and 2019 reports, respectively (2-4). Between 2005 to 2016, neonatal mortality decreased from 39 to 29 deaths per 1000 live births, but in 2019 had stagnated at around 30. Both health system expansion and socio-economic improvements have contributed to the reductions in mortality (5).

In 2003, the Ethiopian government launched the health extension program to increase primary care services (1). Salaried female workers, known as Health Extension Workers (HEWs), were trained to provide basic community-based services. Two HEWs at the health post serve a population of approximately 5000 people. Five health posts and their referral health centre and primary hospital comprise a primary health care unit. The health extension program has 17 packages that fall under four broad areas: family health services, disease prevention and control, hygiene and environmental sanitation, and health education and communication (1). The HEWs are supported by the Women's Development Army leaders, a network of volunteer women established in 2011, who, along with other development goals, promote healthy practices in the community (6, 7).

In 2010, the integrated Community Case Management (iCCM) strategy was launched, allowing HEWs to manage common childhood illnesses in children under five years of age, including pneumonia, malaria, and diarrhoea (8). In 2013, the Community-Based Newborn Care (CBNC) was integrated into the health extension program (9). This initiative enabled HEWs to provide antibiotics for young infants with symptoms of possible severe bacterial infection when a referral was not possible.

Care-seeking for sick under-five children has remained low (10). In 2016, only one-third of children with diarrhoeal diseases received oral rehydration solution, and a similar proportion with suspected pneumonia was taken to an appropriate care provider (2).

For peer review only

For peer review only - http://bmjopen.bmj.com/site/about/guidelines.xhtml

Several barriers to the utilization of services for childhood illnesses have been identified. The perceived quality of services provided by the HEWs, financial constraints, and preference for alternative care providers affected the utilization of child health services (11-13). Service uptake was associated with maternal education levels, parents' knowledge of danger signs, the type of illness, local beliefs, and the need for permission from family decision-makers to seek care (12, 14-16). Health post closure, absence of HEWs, lack of essential drugs and supplies, distance, and the quality of services also contributed to the low service utilization(12, 14, 17, 18). Insufficient supervision and lack of government accountability and ownership of child health services also constituted barriers (8).

Based on a barriers-and-facilitators study, the Ethiopian Government initiated the Optimizing the Health Extension Program (OHEP) intervention (19). OHEP was based on the following hypotheses: community engagement activities would enhance caregiver knowledge and household practices. Further, capacity strengthening would improve the availability of quality services in the CBNC and iCCM programs, and promotion of district-level ownership and accountability would integrate these services into the district level planning and budgeting. These different components together would hypothetically lead to increased utilization of CBNC and iCCM services.

This study aimed to assess the extent to which the OHEP intervention increased care-seeking for children under the age of five years, by comparing changes over time in intervention and comparison areas. Secondary outcomes included treatment of sick children with diarrhoea, suspected pneumonia, or neonatal sepsis.

METHODS

Study setting

The Ethiopian Government initiated the OHEP intervention in 26 districts of Amhara, Southern Nation, Nationalities and Peoples, Oromia, and Tigray regions with an approximate population of 3.5 million

For peer review only - http://bmjopen.bmj.com/site/about/guidelines.xhtml

(Figure 1). The intervention started in 2016 and lasted for a duration of 2.5 years. The implementers were four Non-Governmental Organizations (PATH, UNICEF, Save the Children, and Last 10 Kilometres/John Snow Inc.). The intervention had three components: 1) community engagement, 2) primary care level capacity building, and 3) ownership and accountability of child health services at the district level. The intervention activities under these components, along with the underlying assumptions, intermediate indicators, and outcomes are detailed in Table 1.

Figure 1 Map of Ethiopia showing all regions (left) and the intervention and comparison districts within the four study regions (right).

Table 1 Optimizing the Health Extension Program intervention implemented in 26 districts of Ethiopia, the assumptions and the expected intermediate indicators and outcomes.

Study design

The protocol for the evaluation of the OHEP implementation has been published (20). This study was based on a plausibility design with 26 intervention and 26 comparison districts (woredas) in four regions of Ethiopia. The baseline survey was conducted from December 2016 to February 2017 and the endline survey from December 2018 to February 2019 (Figure 1). The surveys were conducted by the London School of Hygiene and Tropical Medicine and Ethiopian Public Health Institute along with representatives from Gondar, Jimma, Mekelle and Hawassa Universities.

Sample

Intervention districts had a low coverage of maternal, newborn, and child health indicators. Comparison districts were selected by the Regional Health Bureaus to be similar to the size of the population, the burden of diseases, number of primary health care units, health service coverage, length of iCCM and CBNC service delivery, and absence of partners implementing other demand generation activities.

For peer review only - http://bmjopen.bmj.com/site/about/guidelines.xhtml
For peer review only

We used a two-stage stratified cluster sampling to select a representative sample of households within intervention and comparison areas. In the first stage, a list of all enumeration areas of the study districts was obtained based on the 2007 Ethiopian Housing and Population Census. Two hundred enumeration areas (clusters) were selected with probability proportional to the size of the district. Each cluster served as the primary sampling unit. Within clusters, households were selected by systematic random sampling. The Women's Development Army leaders, HEWs, health posts, health centres with staff, and woreda health offices serving the selected clusters were also surveyed.

The sample size was calculated to measure changes with adequate power in a fixed number of percentage points of key indicators between intervention and comparison areas at baseline and endline. Based on the Ethiopian DHS data, a cross-sectional survey of 3000 households in 100 intervention and 100 comparison clusters was expected to find 1747 children under the age of five years in each arm (21). A Tanzanian childhood study found that 50% of under-fives had an illness in the two weeks before the survey (22). The current research assumed more conservatively 30% of children 2-59 months to have had any illness during the two weeks before the interview. This sample size of 3000 households per group with 90% completeness and a design effect of 1.3 would give 80% power to detect differences of 10-20 percentage points across a range of child health indicators (5% significance level). The baseline survey found fewer than the expected number of sick children in the two weeks before the survey. As a result, the household sample size for the endline survey was doubled. We used the baseline list of households to select 66 households randomly in each enumeration area.

Measurements

For every selected household, we interviewed the household head, listed residents, and collected sociodemographic characteristics. The interviews included caregivers of children aged 2-59 months to assess their knowledge of childhood illnesses and care-seeking for sick under-five children in the two

For peer review only - http://bmjopen.bmj.com/site/about/guidelines.xhtml

weeks before the survey. Further, women of reproductive age (13 to 49 years) were interviewed to identify births in the 12 months before the survey, with additional questions on care-seeking for illness in the neonatal period. Up to three visits were made to each participant to ensure the study reaches its target sample size.

The health facility questionnaires assessed the infrastructure, equipment, supplies, and staff available on the day of the survey. Also, data were collected from facility registers on services provided to sick children at health posts and health centres in the three months preceding the survey. The health centre staff, HEWs, and Women's Development Army leader modules covered their background, knowledge, training in the last twelve months, supervision in the previous six months and the services they provided in the last three months. Interviewers collected information at the district health office on demography and characteristics that might affect services for under-five children.

The questions and content of each survey module were based on existing large-scale survey tools and the authors' previous evaluation of the iCCM and CBNC programs (22, 23). All questionnaires were translated into three local languages (Amharic, Oromifa, and Tigrigna), pre-tested and revised. Data collectors and supervisors were trained over ten days, including field training before the start of data collection. They were not provided information on whether a district was an intervention or comparison area.

Data were collected on personal digital assistants (Companion Touch 8), and tablets (Toshiba and Hewlett Packard) programmed with CSPro 6.3 at baseline and CSPro 7.1 at the endline. Data collectors sent encrypted data from the field to the password-protected server at the Ethiopian Public Health Institute. Data managers conducted quality checks and feedback to field teams. Data were cleaned, checked for errors, including consistency and completeness. Since OHEP was a community and health system level intervention, no data monitoring committee was not deemed necessary.

For peer review only - http://bmjopen.bmj.com/site/about/guidelines.xhtml

Outcomes

The primary outcome was the proportion of children 2-59 months old who were reported to have had any illness in the two weeks before the survey for whom advice or treatment was sought from an appropriate provider (health post, health centre, hospital, and private clinic).

Secondary outcomes included: 1) the proportion of sick 2-59 months old children who were reported to have received appropriate treatment for diarrhoea (oral rehydration solution (ORS) with or without zinc tablets) and possible pneumonia (antibiotics), 2) the proportion of infants born in the 12 months before the survey who were reported to have had any illness in the first 28 days of life for whom advice or treatment was sought from an appropriate provider, and 3) the proportion of infants born in the 12 months before the survey who received adequate treatment for suspected neonatal sepsis (antibiotics). Since malaria was not a common illness in the study areas at the time of the surveys, the assessment of appropriate treatment for this illness was excluded. Additionally, the registers for children aged 0-59 days and 2-59 months old were reviewed to assess the median number of children seeking care at health centres and health posts in the three months before the surveys.

We also evaluated intermediate indicators that included, at the community level, the proportion of caregivers that knew signs of illness in children and the proportion that cited appropriate action to be taken for a sick child under five years of age. We also assessed the proportion of caregivers that reported receiving health messages on common childhood illnesses and those attending community meetings to discuss maternal, newborn, and child health issues. At the health system level, we assessed the proportion of HEWs that had received training and the proportion that had attended performance review and clinical mentoring meetings. We also evaluated the proportion of health centres and health posts that had received supervision and the proportion that had the necessary equipment, supplies, and drugs for the provision of child health services. District-level ownership and accountability for child

For peer review only - http://bmjopen.bmj.com/site/about/guidelines.xhtml

health programs were reflected in the proportion of districts that had iCCM and CBNC scorecards. These cards were program management tools for setting targets and monitoring performance. Information was also collected on the average number of ambulances available in districts to transport sick under-five children and whether there were standardized hours of operation for health posts.

Analyses

Descriptive analysis of baseline and endline characteristics in intervention and comparison areas was conducted at the household, caregiver, child, health facility, and district levels. Categorical variables were summarized using percentages with 95% confidence intervals. We used means, with standard errors, or medians, with interquartile ranges, to summarise continuous variables.

At the district level, the demographic and health system level characteristics were examined. For households, the characteristics of mothers or caregivers of children aged 2-59 months, and women who had a delivery in the 12 months before the survey were assessed. Distribution of age, religion, education, self-reported distance to the nearest health post, and socioeconomic status was analysed in intervention and comparison areas at baseline and endline surveys. Similar assessments were done for the distribution of age and sex among children 2-59 months of age and infants born during the 12 months before the survey. Household socioeconomic status was captured by asset ownership, access to utilities, and household characteristics. These were aggregated into a single wealth index score using principal component analysis (24). The household aggregated scores were grouped into wealth quintiles, where quintile 1 represented the poorest fifth of the households, and quintile 5 represented the least poor fifth. A linear or logistic regression model was fitted, depending on the variable type, to assess if there were any differences between intervention and comparison areas that changed over time. A variable was considered a potential confounder if the differences between intervention and comparison areas showed a statistically significant (p-value <0.05) change over time.

For peer review only - http://bmjopen.bmj.com/site/about/guidelines.xhtml

Caregivers' knowledge, practice, and community participation relating to child health were assessed. Further, the health system level factors associated with child health services, including training and supervision of health workers providing under-five services, and the observed availability of infrastructure, equipment, supplies, and drugs for the treatment of childhood illnesses at health posts and health centres were assessed.

Using data from facility registers, we also compared the median number of young infants and children 2-59 months of age who received care in the three months before the survey in intervention and comparison areas at baseline and endline. We analysed for differences between intervention and comparison areas over time using quantile regression analysis.

Difference-in-differences analyses were used to estimate the effect of the OHEP intervention on care-seeking for sick under-five children. Binary outcome indicators were used to capture whether a sick child had sought care or received treatment. The key independent variable for the outcomes of this study was whether the child lived in the OHEP intervention or comparison area. A model was then created that included an interaction term for the timing of the survey (baseline or endline) and the survey area (OHEP intervention or comparison area). This model allowed for the calculation of the odds of care-seeking or treatment for under-five children in intervention areas as compared to comparison areas, accounting for any differences between baseline and endline survey areas, with adjustment for the cluster sampling and identified confounding factors. The Stata 13 (Stata Corporation, College Station, Texas) svy commands were used to adjust for clustering. The assessment used a blinded analysis. The code identifying the intervention and comparison areas were revealed after the analysis and interpretations were completed.

For peer review only - http://bmjopen.bmj.com/site/about/guidelines.xhtml

Patient and public involvement

Patient and/or the public were not involved in the design or conduct, or reporting or dissemination plans of this research.

RESULTS

The OHEP intervention was fully operational from the start of 2017 until October 2018. A process evaluation of the implementation will be reported elsewhere (Okwaraji et al., in preparation). Briefly, a majority of the activities were one-off, assuming that health extension workers would catalyse these activities in the communities. The fidelity of the 31 activities varied by district and over time. Many activities were delayed to the last year. In eight of the districts, implementation activities were interrupted 4 months for administrative reasons. Social and political unrest in some of the districts had an impact on health services.

Out of the 200 clusters eligible, six intervention clusters were excluded at baseline due to security reasons and, therefore, not included in the endline survey (Figure 2). At endline, a further three intervention clusters and ten comparison clusters were excluded due to security issues.

Figure 2 Study flow diagram of intervention and comparison categorised by baseline and endline data collection period.

District-level data were not available from five intervention and one comparison districts at baseline, while at endline data were missing from four intervention and two comparison districts (S1 Table). The demographic and health systems characteristics of intervention and comparison districts at baseline and endline surveys were similar. The study districts had an average population size of 130,000 inhabitants, with 23% being women 15-49 years, 20% being children below the age of five years. The average household size was 4.6 persons. One-third of the districts had a hospital. There were, on average, five

For peer review only - http://bmjopen.bmj.com/site/about/guidelines.xhtml

health centres per district and five health posts under each health centre. At the time of the baseline survey, there were 1-2 ambulances available on average in the district for transporting sick children, while this had increased to 2-3 at the time of the endline survey. There were also some increases in staffing; health officers at the health centres increased from 2 to 2.5, midwives at health centres from 2.5 to more than 3, and the number of HEWs increased from an average slightly above 2 per health post to almost 3. These characteristics were similar across intervention and comparison districts. At the time of the baseline survey, CBNC and iCCM indicators were included in the scorecards of 79% of intervention and over 90% of comparison districts. At the endline, over 90% of all districts had iCCM and CBNC indicators in their scorecards.

The distribution of study participants by age, education, and socio-economic status across intervention and comparison areas showed no evidence of a change over time. There was some evidence that the proportion of Orthodox Christians changed over time, as did the reported distance to the nearest health post. Both were included as confounders in the analysis of the primary outcome. The distribution of sex and age of under-five children remained similar in the study areas over time (Table 2). The characteristics of women who had delivered in the 12 months before the survey showed no evidence of change over time. Young infant age distribution changed, which was included as a potential confounder in the analyses (Table 3).

Table 2 Characteristics of mothers or caregivers of children aged 2-59 months and of their children at baseline (December 2016- February 2017) and endline surveys (December 2018-February 2019) in intervention and comparison areas.

Table 3 Characteristics of mothers and their children born in the 12 months prior to the survey at baseline (December 2016- February 2017) and endline surveys (December 2018-February 2019) in intervention and comparison areas.

For peer review only - http://bmjopen.bmj.com/site/about/guidelines.xhtml

There was no association between the intervention and child health messages the caregivers had received or attendance at community health-related meetings (S2 Table). Between baseline and endline, caregivers' unprompted knowledge on sick newborn danger signs, such as limited or no movement and skin pustules, showed some increase in intervention areas as compared to comparison areas over time (S3 Table). At baseline and endline, over four-fifths of the caregivers in intervention as well as comparison areas said they would take their child to the health centre for a range of childhood illnesses, while approximately one-fifth said they would go to a health post (S4 Table).

Two or more HEWs were available in most health posts without association to the intervention (p=0.55 in difference-in-differences analysis) (S5 Table). Most health posts were open five days a week without any association with the intervention (p=0.99 in difference-in-differences analysis). The proportion of health posts that posted their operational days and hours decreased in both areas over time.

The proportion of HEWs trained in iCCM and CBNC remained similar at baseline and endline in intervention and comparison areas (Table 4). Maternal, newborn, and child health orientation for Women's Development Army leaders decreased slightly in intervention areas and had a minor increase in comparison areas (p=0.34 in difference-in-differences analysis).

Table 4 Child health training for health extension workers and Women's Development Army leaders at baseline (December 2016- February 2017) and endline surveys (December 2018-February 2019) in intervention and comparison areas

At baseline, approximately two-thirds of health centre staff in the intervention and comparison areas reported receiving a supervisory visit in the last three months; this remained similar at endline (S6 Table). Over half of all the surveyed HEWs at baseline and endline reported receiving a supervisory visit in the last month. Approximately half in both areas and at both time points reported attending a performance review and clinical mentoring meeting in the six months before the survey. One in six Women's Development Army leaders reported meeting with HEWs and other leaders in the three

For peer review only - http://bmjopen.bmj.com/site/about/guidelines.xhtml

months prior to the survey in intervention and comparison areas at baseline and remained similar over time.

Almost all health centres and most health posts had patient toilets (Table 5). Water availability on the day of the survey decreased over time in intervention area health centres and health posts, and at endline about three-quarters of health centres and half of the health posts in both study areas had water. Electricity on the day of the survey was available in approximately two-thirds of the health centres and one-fifth of the surveyed health posts at baseline and endline.

Table 5: Observed availability of infrastructure, equipment, supplies and drugs for treatment of childhood illness at health posts and health centres at baseline (December 2016- February 2017) and endline surveys (December 2018-February 2019) in intervention and comparison areas

At baseline and endline, antibiotics needed to treat sick under-five children were available in most health centres (Table 5). At health posts, over three-quarters had amoxicillin. However, gentamicin was not available in over half of the intervention and comparison area health posts at baseline and endline. ORS and zinc were available in almost all health centres at both time points. Zinc was also available in most health posts at both time points. ORS availability increased over time in both intervention and comparison areas. Almost all health centres had job aids and equipment needed for managing sick under-five children. Health posts lacked some equipment in both study areas at baseline and endline, while most had the necessary job aids at both time points.

The median number of sick young infants registered in intervention area health centres in the quarter before the survey at baseline was five and increased to nine at the endline, while the median remained at three for comparison areas health centres (p=0.08) (Table 6). Intervention area health centres at baseline and endline had registered a similar median number of sick 2-59 months old children (237 vs. 232), while comparison area health centres registered fewer (149 vs. 128) median number of cases (p=0.58). The median number of sick young infants seen at intervention and comparison area health posts for the quarter before the survey was zero at both baseline and endline. The median number of children

For peer review only - http://bmjopen.bmj.com/site/about/guidelines.xhtml

2-59 months seen increased from 18 to 22 in intervention areas and decreased from 13 to 10 in comparison areas (p=0.22).

Table 6 Median number of newborns (0-2 months) and children (2-59 months) registered in health centres and health posts in the quarter prior to survey at baseline (December 2016- February 2017) and endline surveys (December 2018-February 2019) in intervention and comparison areas.

At baseline, 6% of intervention and 5% of comparison area caregivers reported that their 2-59 months old children had been sick in the two weeks before the survey (Table 7). At endline, reported illness had increased (13% and 11%). Care-seeking for any illness at baseline in intervention areas was 58% and 49% in comparison areas, while at endline it was 39% in intervention areas and 34% in comparison areas (difference-in-differences -4 percentage points, adjusted odds ratio (AOR)=0.49 (95% CI: 0.12-1.95)). The use of ORS with zinc for diarrhoea was 32% in the intervention areas and 25% in comparison areas at baseline, while at endline it was to 38% in intervention areas and 30% in comparison areas (difference-in-differences +1, AOR =0.70 (95% CI: 0.04-13.66)). The use of ORS with or without zinc was higher in intervention than in comparison areas (53% vs. 40%) at baseline, while at endline it was similar (50% vs. 53%) in both areas (difference-in-differences -16, AOR = 0.29 (95% CI: 0.02-5.33)). Antibiotic treatment for reported signs and symptoms of possible pneumonia in the intervention areas was higher than in comparison areas at baseline (67% vs. 56%). At endline, it was 62% in intervention and 69% in comparison areas (difference-in-differences -18, AOR =0.15 (95% CI:0.00-16.18)).

Table 7 Illness, care-seeking and case-management in the two weeks prior to the survey among children 2-59 months of age reported by caregivers from the household survey at baseline (December 2016- February 2017) and endline surveys (December 2018-February 2019) in intervention and comparison areas.

Among those that reported illness in the neonatal period, over 70% reported signs and symptoms of possible sepsis (Table 8). At baseline care-seeking for any neonatal illness was higher in intervention as

For peer review only - http://bmjopen.bmj.com/site/about/guidelines.xhtml

compared to comparison areas (74% vs. 44%), while at endline it was lower (51% vs. 68%) in the intervention than in comparison areas (difference-in-differences -47, AOR =0.04 (95% CI: 0.00-0.60)). Antibiotic treatment among those with possible sepsis at baseline was 51% in the intervention and 36% in comparison areas and. At endline, it was 57% in the intervention and 68% in comparison areas (difference-in-differences -26, AOR =0.19 (95% CI: 0.02-2.42)).

Table 8 Morbidity, care-seeking and management among infants in the neonatal period at baseline (December 2016- February 2017) and endline surveys (December 2018-February 2019) in intervention and comparison areas.

DISCUSSION

This study found that the Optimizing the Health Extension Program intervention neither had any effect on care-seeking for any illness nor on treatment for diarrhoea or possible pneumonia in children 2-59 months of age. Neither did we find an evidence of an effect on care-seeking for neonatal illness nor on the treatment of possible severe bacterial neonatal infection. These findings were based on household surveys and were supported by results from register reviews at health posts and health centres that showed a low level of service utilization for sick under-five children at baseline and endline surveys. The intervention did not affect caregivers' participation in community engagement activities. No changes were observed in facility preparedness in health centres and health posts that could be related to the intervention. The health system characteristics at the district level showed small changes, which were not associated with the intervention.

The OHEP intervention took place in selected districts of four regions of Ethiopia. Study population characteristics in intervention and comparison districts were broadly similar. A few of the household characteristics differed between intervention and comparison areas but had a marginal influence on the analyses. It is, however, possible that unmeasured confounders might contribute to the observed

For peer review only - http://bmjopen.bmj.com/site/about/guidelines.xhtml
For peer review only

results. Given that child morbidity has a seasonal variation, both surveys were done during the same months of the year. At baseline, the overall proportion of reported illness in the last two weeks was lower than anticipated, and this was higher at the endline. The difference in the reported proportion of sick children could be due to differences in the interaction and the extent of the probing between data collectors and families when enquiring about childhood illnesses. Field interviewers were, however, blinded to the allocation, i.e., whether the district where they collected data was an intervention or comparison area. Any differential reporting of care utilization between intervention and comparison areas is, therefore, unlikely. Service utilisation records from health posts and health centres supported the household-level findings of no change in service utilization as a result of the intervention.

The three components of the OHEP intervention were based on a logic framework, which was developed from an analysis of barriers to child health service utilization in Ethiopia (19). As one of the components, OHEP included several strategies to engage community members, which have been reported to be effective in Ethiopia and elsewhere (25-27). The OHEP intervention did not influence caregivers' community engagement related to maternal, newborn, and child health issues. One plausible explanation for the lack of observed effect in the present study may be the relatively short duration of the intervention (28). In comparison with single or multi-component intervention, complex social and health systems interventions, such as OHEP, should not be expected to result in rapid effects on care utilization (29). It is also possible that the interaction between different components in a complex intervention within a particular setting might introduce unpredictable effects (30).

The second component of OHEP, primary care capacity building, included training of health workers and volunteers, supportive supervision, and performance review and clinical mentoring. No changes were observed in the iCCM and CBNC training of HEWs that could be related to the intervention. Training alone may not be sufficient to improve health care provider's performance (31). Studies conducted in Ethiopia on supportive supervision and performance review and clinical mentorship meetings have also

For peer review only - http://bmjopen.bmj.com/site/about/guidelines.xhtml

shown that these can be effective means of improving services provided by HEWs (32). The OHEP intervention had no effect on the supervision and the performance review and clinical mentoring meetings provided for HEWs. The quality of iCCM services measured at baseline was low, and it is unlikely to have been improved by the training, supervision, and infrequent clinical mentoring supported by OHEP (18).

It is essential to ensure that the actors who are involved in the provision of childhood services are accountable in the development, financing, implementation, and monitoring of the programs (33). The third component of OHEP addressed district level ownership and accountability through several means, which included availability of ambulances for child referral and inclusion of indicators to monitor iCCM and CBNC service delivery. Although more ambulances were available at the endline, the increase was not linked to the intervention. Almost all intervention and comparison districts at the endline had indicators to monitor iCCM and CBNC at the district level.

In the current study, approximately half of the ill children 2-59 months of age sought care at baseline, and this decreased to one-third at the endline. Other studies have reported similar and even lower levels of iCCM service uptake in Ethiopia (2, 14, 34). Our baseline result for care-seeking was higher than expected, while the endline proportion follows the gradually increasing trend seen in the DHS surveys (2, 4, 21). Treatment for diarrhoea estimated in this study seemed to follow a plausible upward trend seen across the DHS. The estimated antibiotic treatment for possible pneumonia, which was less than 10% in 2005 and 2011 DHS, was two-thirds in our study. It should be noted that household surveys may not provide a reliable estimate of treatment for possible pneumonia (35, 36).

Almost all health centres had ORS, amoxicillin, and gentamicin, and nearly all health posts had ORS. Some health posts, however, did not have amoxicillin, and most did not have gentamicin. Compared to a recent study done in Ethiopia, amoxicillin availability in the current study was higher, while the

For peer review only - http://bmjopen.bmj.com/site/about/guidelines.xhtml

availability of gentamycin was similar (8). This lack of appropriate antibiotics has implications for the services provided to sick under-five children, particularly services for neonates. Most caregivers also indicated that they would take their sick child to a health centre rather than to a health post. Stock-out of drugs, health post closure, lack of knowledge on the type of available services, and the acceptability and quality of the services provided by HEWs might contribute to caregivers' preference for health centres (12-14).

It is important to consider the context in which OHEP was implemented to understand the observed outcomes in this evaluation. OHEP had many sequential activities, requiring a long time to implement all the components. In some districts, many intervention activities were only fully implemented in the second year, which was late in the project period. Some of the intervention activities, such as the health post open house, were one-off efforts that were unlikely to have a sustained effect on service utilization. The assumptions under which the OHEP intervention was expected to succeed, for example, community influencers becoming change agents to promote child health services, might not have been met. The lack of drugs, particularly for CBNC services, also indicates that the assumption that the public sector and supply chain partners would ensure drug availability was not fully met. In some districts, implementation was also interrupted for several months, either due to political instability or project-related delays.

CONCLUSION

This evaluation in four regions of Ethiopia showed that the OHEP intervention did not have an effect on care-seeking for sick under-five children. The lack of effect could be attributed to the relatively short period of OHEP implementation, the nature and unmet assumptions of the intervention, and implementation interruption. Future funding schemes need to take into consideration that complex interventions with multiple components, including behaviour change, need a more extended

For peer review only - http://bmjopen.bmj.com/site/about/guidelines.xhtml

implementation period to measure the effectiveness of the program. This evaluation is linked to an ongoing process evaluation as well as in-depth sub-studies that address the quality of care provided by HEWs, equity, and geographic distribution of service utilization that can also offer further explanations to the observed lack of effect (18, 37, 38).

Authors' contributions

The study was conceived by JAS and ETL with inputs from DB, YO, AD, ABB, AAM, MY, MA, FWB, AD, FA, DWD, HBo, HBe, AT, and LP. All authors contributed to acquisition of the data and DW was the data manager. JS, LP, YO, DB and AD analysed and interpreted the data. DB prepared the first draft of the manuscript with contributions from JS, LP, YO and AD. All authors read and commented on the manuscript and approved the final version.

Acknowledgements

The authors would like to thank Lindsay Mangham-Jefferies for drafting the initial protocol and questionnaires for the surveys. The authors would also like to thank the field teams that were involved in the data collection as well as the government official that facilitated the administration of the surveys. They also thank the study participants who agreed to generously give their time to participate in study.

Ethical considerations

Ethical approvals were obtained from the Ethiopian Public Health Institute (Ethics Ref 613/52) and the London School of Hygiene & Tropical Medicine (Ethics Ref 16117). Information sheets translated into the three local languages were read out to study participants to obtain their written informed consent.

For peer review only

For peer review only - http://bmjopen.bmj.com/site/about/guidelines.xhtml

Data availability statement

Request for data can be made to Della Berhanu (della.berhanu@lshtm.ac.uk). A data sharing committee has been established. All requests will be reviewed by this committee and if granted, data will be shared without any identifiers.

Funding

This project was funded by Bill & Melinda Gates Foundation (OPP1132551). The funder had no role in the study design, collection, management, analysis, or interpretation of data.

Competing interests

The authors declare that they have no competing interests.

For peer review only

For peer review only - http://bmjopen.bmj.com/site/about/guidelines.xhtml

REFERENCES

1. Assefa Y, Gelaw YA, Hill PS, Taye BW, Van Damme W. Community health extension program of Ethiopia, 2003-2018: successes and challenges toward universal coverage for primary healthcare services. Global Health. 2019;15(1):24.
2. International. CSAaI. Ethiopia Demogrpahic and Health Survey 2016. Addis Ababa, Ethiopia and Rockville, Maryland, USA: 2016. 2016.
3. International. CSAaI. Ethiopian Demogrpahic and Health Survey 2019. Addis Ababa, Ethioia and Calvertn Marlyand, USA: . 2019.
4. International. CSAaI. Ethiopian Demogrpahic and Health Survey 2005. Addis Ababa, Ethiopia and Rockville, Maryland, USA. 2006.
5. Ruducha J, Mann C, Singh NS, Gemebo TD, Tessema NS, Baschieri A, et al. How Ethiopia achieved Millennium Development Goal 4 through multisectoral interventions: a Countdown to 2015 case study. Lancet Glob Health. 2017;5(11):e1142-e51.
6. Yitbarek K, Abraham G, Morankar S. Contribution of women's development army to maternal and child health in Ethiopia: a systematic review of evidence. BMJ Open. 2019;9(5):e025937.
7. Damtew ZA, Karim AM, Chekagn CT, Fesseha Zemichael N, Yihun B, Willey BA, et al. Correlates of the Women's Development Army strategy implementation strength with household reproductive, maternal, newborn and child healthcare practices: a cross-sectional study in four regions of Ethiopia. BMC Pregnancy Childbirth. 2018;18(Suppl 1):373.
8. Hailemariam S, Gebeyehu Y, Loha E, Johansson KA, Lindtjorn B. Inadequate management of pneumonia among children in South Ethiopia: findings from descriptive study. BMC Health Serv Res. 2019;19(1):426.
9. Mathewos B, Owen H, Sitrin D, Cousens S, Degefie T, Wall S, et al. Community-Based Interventions for Newborns in Ethiopia (COMBINE): Cost-effectiveness analysis. Health Policy Plan. 2017;32(suppl_1):i21-i32.
10. Amouzou A, Hazel E, Shaw B, Miller NP, Tafesse M, Mekonnen Y, et al. Effects of the integrated Community Case Management of Childhood Illness Strategy on Child Mortality in Ethiopia: A Cluster Randomized Trial. Am J Trop Med Hyg. 2016;94(3):596-604.
11. Tefera W, Tesfaye H, Bekele A, Kayessa E, Waltensperger KZ, Marsh DR. Factors influencing the low utilization of curative child health services in Shebedino District, Sidama Zone, Ethiopia. Ethiop Med J. 2014;52 Suppl 3:109-17.
12. Shaw B, Amouzou A, Miller NP, Tafesse M, Bryce J, Surkan PJ. Access to integrated community case management of childhood illnesses services in rural Ethiopia: a qualitative study of the perspectives and experiences of caregivers. Health Policy Plan. 2016;31(5):656-66.
13. King R, Jackson R, Dietsch E, Hailemariam A. Barriers and facilitators to accessing skilled birth attendants in Afar region, Ethiopia. Midwifery. 2015;31(5):540-6.
14. Shaw B, Amouzou A, Miller NP, Tsui AO, Bryce J, Tafesse M, et al. Determinants of Utilization of Health Extension Workers in the Context of Scale-Up of Integrated Community Case Management of Childhood Illnesses in Ethiopia. Am J Trop Med Hyg. 2015;93(3):636-47.
15. Nigatu SG, Worku AG, Dadi AF. Level of mother's knowledge about neonatal danger signs and associated factors in North West of Ethiopia: a community based study. BMC Res Notes. 2015;8:309.
16. Degefie T, Amare Y, Mulligan B. Local understandings of care during delivery and postnatal period to inform home based package of newborn care interventions in rural Ethiopia: a qualitative study. BMC Int Health Hum Rights. 2014;14:17.
17. Tilahun H, Fekadu B, Abdisa H, Canavan M, Linnander E, Bradley EH, et al. Ethiopia's health extension workers use of work time on duty: time and motion study. Health Policy Plan. 2017;32(3):320-8.

For peer review only - http://bmjopen.bmj.com/site/about/guidelines.xhtml

18. Getachew T, Mekonnen S, Yitayal M, Persson LA, Berhanu D. Health Extension Workers' diagnostic accuracy for common childhood illnesses in four regions of Ethiopia: a cross-sectional study. Acta Paediatr. 2019.
19. UNICEF P. Literature Review on Barriers to Utilization of Health Extension Services: Draft Report 2016.
20. Berhanu D, Okwaraji YB, Belayneh AB, Lemango ET, Agonafer N, Birhanu BG, et al. Protocol for the evaluation of a complex intervention aiming at increased utilisation of primary child health services in Ethiopia: a before and after study in intervention and comparison areas. BMC Health Serv Res. 2020;20(1):339.
21. International. CSAaI. Ethiopian Demogrpahic and Health Survey 2011. Addis Ababa, Ethioia and Calvertn Marlyand, USA: . 2012.
22. Armstrong Schellenberg JR, Adam T, Mshinda H, Masanja H, Kabadi G, Mukasa O, et al. Effectiveness and cost of facility-based Integrated Management of Childhood Illness (IMCI) in Tanzania. Lancet. 2004;364(9445):1583-94.
23. Berhanu D, Avan BI. Community Based Newborn Care: Baseline report summary, Ethiopia Octboer 2014. London: IDEAS London School of Hygiene & Tropical Medicine. 2014. . 2014.
24. Vyas S, Kumaranayake L. Constructing socio-economic status indices: how to use principal components analysis. Health Policy Plan. 2006;21(6):459-68.
25. Barry D, Frew AH, Mohammed H, Desta BF, Tadesse L, Aklilu Y, et al. The effect of community maternal and newborn health family meetings on type of birth attendant and completeness of maternal and newborn care received during birth and the early postnatal period in rural Ethiopia. J Midwifery Womens Health. 2014;59 Suppl 1:S44-54.
26. Tesfaye S, Barry D, Gobezayehu AG, Frew AH, Stover KE, Tessema H, et al. Improving coverage of postnatal care in rural Ethiopia using a community-based, collaborative quality improvement approach. J Midwifery Womens Health. 2014;59 Suppl 1:S55-64.
27. Evaluation WHOaIIfI. An evidence map of social, behavioural and community engagement interventions for reproductive, maternal, newborn and child health. Geneva: World Health Organization. 2017.
28. Persson LA, Nga NT, Malqvist M, Thi Phuong Hoa D, Eriksson L, Wallin L, et al. Effect of Facilitation of Local Maternal-and-Newborn Stakeholder Groups on Neonatal Mortality: Cluster-Randomized Controlled Trial. PLoS Med. 2013;10(5):e1001445.
29. Osrin D, Prost A. Perinatal interventions and survival in resource-poor settings: which work, which don't, which have the jury out? Arch Dis Child. 2010;95(12):1039-46.
30. Hawe P. Lessons from complex interventions to improve health. Annu Rev Public Health. 2015;36:307-23.
31. Rowe AK, Rowe SY, Peters DH, Holloway KA, Chalker J, Ross-Degnan D. Effectiveness of strategies to improve health-care provider practices in low-income and middle-income countries: a systematic review. Lancet Glob Health. 2018;6(11):e1163-e75.
32. Miller NP, Amouzou A, Hazel E, Legesse H, Degefie T, Tafesse M, et al. Assessment of the impact of quality improvement interventions on the quality of sick child care provided by Health Extension Workers in Ethiopia. J Glob Health. 2016;6(2):020404.
33. George A, Rodríguez DC, Rasanathan K, Brandes N, Bennett S. iCCM policy analysis: strategic contributions to understanding its character, design and scale up in sub-Saharan Africa. Health Policy and Planning. 2015;30(suppl_2):ii3-ii11.
34. Amouzou A, Kanyuka M, Hazel E, Heidkamp R, Marsh A, Mleme T, et al. Independent Evaluation of the integrated Community Case Management of Childhood Illness Strategy in Malawi Using a National Evaluation Platform Design. Am J Trop Med Hyg. 2016;94(3):574-83.

For peer review only - http://bmjopen.bmj.com/site/about/guidelines.xhtml

35. Campbell H, El Arifeen S, Hazir T, O'Kelly J, Bryce J, Rudan I, et al. Measuring coverage in MNCH: challenges in monitoring the proportion of young children with pneumonia who receive antibiotic treatment. PLoS Med. 2013;10(5):e1001421.
36. Hazir T, Begum K, El Arifeen S, Khan AM, Huque MH, Kazmi N, et al. Measuring coverage in MNCH: a prospective validation study in Pakistan and Bangladesh on measuring correct treatment of childhood pneumonia. PLoS Med. 2013;10(5):e1001422.
37. Wuneh AD, Medhanyie AA, Bezabih AM, Persson LA, Schellenberg J, Okwaraji YB. Wealth-based equity in maternal, neonatal, and child health services utilization: a cross-sectional study from Ethiopia. Int J Equity Health. 2019;18(1):201.
38. Defar A, Okwaraji YB, Tigabu Z, Persson LA, Alemu K. Geographic differences in maternal and child health care utilization in four Ethiopian regions; a cross-sectional study. Int J Equity Health. 2019;18(1):173.

For peer review only

For peer review only - http://bmjopen.bmj.com/site/about/guidelines.xhtml

Table 1 Optimizing the Health Extension Program intervention implemented in 26 districts of Ethiopia, the assumptions and the expected intermediate indicators and outcomes.

Assumptions		
• Health managers and political leaders at all levels will be committed to supporting the intervention<br>• Strong coordination and partnership among the stakeholders at all levels<br>• Community influencers (religious and traditional leaders) will be change agents in promoting maternal, newborn and child health services<br>• Public sector and supply chain partners will ensure drug and service availability		
Intervention		
Community engagement	Primary care level capacity building	Ownership and accountability
• Health post open house<br>• Group discussions led by Women's Development Army leaders<br>• Engaging schools<br>• Engaging religious and traditional leaders<br>• Engaging agricultural extension workers, to reach male partners<br>• Educational health films<br>• Radio spots<br>• Printed information and education communication materials	**Women's Development Army:**<br>• Level one training<br>• Provision of job aids and tools<br>• Community based data for decision-making training for Women's Development Army leaders<br><br>**Health Extension Workers:**<br>• Gap-filling training and job aids<br>• Supportive supervision<br>• Performance review and clinical mentoring meetings<br>• Community based data for decision making training of trainers for Health Extension Workers	• Advocacy for the integration of Community-Based Newborn Care and integrated Community Case Management into planning, budgeting, management, and information systems<br>• District level annual based planning<br>• Management standards for health post opening hours<br>• Ambulance service for children's referral<br>• Engage kebele (sub-district) command post in the efforts<br>• Community forum<br>• Establish community feedback mechanisms
Intermediate indicators		
Caregivers knowledge, practices and community participation on matters relating to child health	Facility readiness in terms of medicine, equipment and supplies as well as health worker training and supervision necessary to provide child health services	Planning and monitoring of child health services in the district health system (availability of ambulance, Community Based Newborn Care and integrated Community Case Management indicators, and standardized operational hours for health posts).
Outcomes		
1. Primary outcome<br>Service utilization from an appropriate provider for sick children between the ages of 2-59 months<br>2. Secondary outcomes<br>Appropriate treatment for diarrhoea and pneumonia<br>Care-seeking for infants who were ill in the neonatal period<br>Appropriate treatment for possible severe bacterial infection in the neonatal period		

For peer review only

For peer review only - http://bmjopen.bmj.com/site/about/guidelines.xhtml

Table 2 Characteristics of mothers or caregivers of children aged 2-59 months and of their children at baseline (December 2016- February 2017) and endline surveys (December 2018-February 2019) in intervention and comparison areas.

	Baseline household survey				Endline household survey				
	Intervention		Comparison		Intervention		Comparison		
	N	% (95%CI)	N	% (95%CI)	N	% (95%CI)	N	% (95%CI)	P-value[a]
**Caregiver's characteristic**									
**Age (years)**	**1259**		**1273**		**2454**		**2275**		
<25		24(22, 27)		27(25, 30)		17(16, 19)		20(19, 22)	0.15[b]
25-29		28(26, 31)		30(27,33)		28(26, 29)		26(25, 28)	
30-34		19(17, 22)		17(15, 19)		23(21, 24)		21(19, 23)	
35-39		16(14, 19)		16(14, 18)		19(17, 21)		18(16, 19)	
>=40		12(10, 14)		10(8, 12)		13(12, 15)		14(13, 16)	
**Education**	**1259**		**1273**		**2454**		**2275**		
Schooling		36(34,39)		45(43, 48)		37(35,39)		45(42,47)	0.07[c]
No schooling		64(61, 66)		55(52, 57)		63(61, 65)		55(53, 58)	
**Religion**	**1259**		**1273**		**2454**		**2275**		
Orthodox Christians		54(51, 57)		38(36, 41)		61(59, 63)		47(45, 49)	0.04[c]
Others[d]		46(43, 49)		62(59, 64)		38(37, 41)		53(51, 55)	
**Socio-economic status**	**1259**		**1273**		**2454**		**2275**		0.81[b]
Q1(poorest)		19(17, 22)		23(21, 25)		18(17, 20)		25(23, 27)	
Q2		18(16, 21)		20(18, 22)		19(18, 20)		22(21, 25)	
Q3		20(18, 23)		18(16, 21)		19(18, 21)		18(16, 19)	
Q4		22(20, 25)		21(18, 23)		20(19, 22)		17(16, 19)	
Q5 (least poor)		19(18, 22)		18(16, 20)		23(22, 25)		17(16, 19)	
**Distance to the nearest HP**	**1104**		**1126**		**2030**		**1878**		<0.01[c]
<=30mins		59(56, 62)		71(69, 74)		64(62, 66)		64(62, 67)	
>30mins		40(39, 44)		29(26,31)		36(34, 38)		36(33,38)	
**Child 2-59 months of age**									
**Sex**	**1525**		**1471**		**2960**		**2740**		
Male		52(49,54)		51(48, 54)		52(50,53)		51(49,52)	0.77[c]
Female		48(46,51)		49(46,51)		48(47,50)		49(48,51)	
**Age (months)**	**1488**		**1453**		**2960**		**2740**		
2-11		19(17,21)		18(16, 20)		16(14,17)		15(14,16)	0.43[b]
12-23		19(17,21)		20(18, 22)		18(17,20)		16(15,18)	
24-35		23(21,25)		22(20, 24)		20(19,21)		19(18,20)	
36-47		24(21,26)		26(24, 28)		27(26,29)		27(26,29)	
48-59		16(14,18)		14(13, 16)		19(17,20)		22(21,24)	

[a] All models were adjusted for clustering
[b] P-value obtained from linear regression model for the variable to assess whether there was any difference between the groups that changed over time.
[c] P-value obtained from logistic regression model for the variable to assess whether there was any difference between the groups that changed over time.
[d] Includes Catholics, Muslims and Protestants.

For peer review only - http://bmjopen.bmj.com/site/about/guidelines.xhtml

Table 3 Characteristics of mothers and their children born in the 12 months prior to the survey at baseline (December 2016- February 2017) and endline surveys (December 2018-February 2019) in intervention and comparison areas.

	**Baseline household survey**				**Endline household survey**				
	Intervention		Comparison		Intervention		Comparison		
	N	% (95%CI)	N	% (95%CI)	N	% (95%CI)	N	% (95%CI)	P-value[a]
**Mother's characteristic**									
**Age (years)**	**331**		**331**		**482**		**400**		
<25		34(29, 40)		34(29, 39)		25(21, 29)		29(25, 34)	0.73[b]
25-29		37(3, 43)		38(32,43)		37(33, 42)		33(29, 38)	
30-34		16(13, 21)		15(12, 19)		22(19, 26)		22(18, 27)	
>=35		12(9, 16)		13(10, 17)		16(13, 20)		16(12, 20)	
**Education**	**331**		**331**		**482**		**400**		0.41[c]
Schooling		42(36, 47)		50(45, 56)		44(40, 49)		54(49, 59)	
No schooling		58(53, 64)		50(44, 55)		56(51, 60)		46(41, 51)	
**Religion**	**331**		**331**		**482**		**400**		
Orthodox Christians		47(41, 52)		32(27, 37)		57(52, 61)		41(36, 46)	0.22[c]
Others[d]		53(48, 52)		68(63, 73)		43(39, 48)		59(54, 64)	
**Socio-economic status**	**331**		**331**		**482**		**400**		
Q1(poorest)		21(17, 26)		21(17, 26)		21(17, 25)		29(25, 34)	0.29[b]
Q2		20(16, 25)		18(14, 23)		22(18,26)		2(18, 26)	
Q3		22(18, 27)		17(14, 22)		18(15, 22)		16(13, 20)	
Q4		17(13, 21)		21(16, 26)		19(15, 22)		19(11, 23)	
Q5(least poor)		20(16, 25)		22(18, 27)		21(17, 25)		14(13, 18)	
**Distance to the nearest HP**	**304**		**303**		**413**		**343**		0.16[c]
<=30mins		60(54, 65)		69(64, 74)		61(56, 66)		66(61, 71)	
>30mins		40(35, 46)		31(26, 36)		39(34, 44)		34(29, 39)	
**Child born in the last 12 months**									
**Sex**	**324**		**328**		**482**		**399**		
Male		49(43,54)		52(46, 57)		55(50,59)		52(47,57)	0.64[c]
Female		51(46,57)		48(43,54)		45(41, 50)		48(43,53)	
**Age (months)**	**331**		**331**		**482**		**400**		
0-2		21(17,26)		26(22,31)		22(18,25)		22(19,27)	0.02[b]
3-5		24(20,29)		32(27, 37)		25(21,29)		24(20,28)	
6-8		30(25,35)		23(18, 28)		26(23,30)		29(25,33)	
9-11		25(21,30)		19(15, 24)		27(23,31)		25 (21,29)	

[a] All models were adjusted for clustering

[b] P-value obtained from linear regression model for the variable to assess whether there was any difference between the groups that changed over time.

[c] P-value obtained from logistic regression model for the variable to assess whether there was any difference between the groups that changed over time.

[d] Includes Catholics, Muslims and Protestants.

For peer review only - http://bmjopen.bmj.com/site/about/guidelines.xhtml

Table 4 Child health training for health extension workers and Women's Development Army leaders at baseline (December 2016- February 2017) and endline surveys (December 2018-February 2019) in intervention and comparison areas

	**Baseline frontline worker survey**		**Endline frontline worker survey**			
	Intervention	Comparison	Intervention	Comparison	Difference in Difference[a]	P-value[b]
**Training**	% (95%CI) [n]	% (95%CI) [n]	% (95%CI) [n]	% (95%CI) [n]	%	
iCCM[c] for health extension workers	83 (74-90) [145]	82 (74-88) [131]	78 (69-85) [141]	80 (70-87) [133]	-3	0.63
CBNC[d] for health extension workers	64 (54-73) [145]	66 (56-74) [130]	69 (61-76) [141]	69 (60-77) [133]	2	0.89
MNCH[e] orientation for Women's Development Army leaders	70 (60-74) [93]	59 (48-68) [94]	62 (53-70) [169]	61 (52-70) [167]	-10	0.34

[a] Difference-in-Difference: the difference in the proportion between intervention and comparison areas at endline subtracted from the difference in proportion between intervention and comparison at baseline.

[b] P-value obtained from a logistic regression model for the difference-in-difference analysis

[c] iCCM: integrated community case management training

[d] CBNC: community-based newborn care management training

[e] MNCH: maternal, newborn, and child health orientation in the last 12 months

For peer review only

For peer review only - http://bmjopen.bmj.com/site/about/guidelines.xhtml

Table 5: Observed availability of infrastructure, equipment, supplies and drugs for treatment of childhood illness at health posts and health centres at baseline (December 2016- February 2017) and endline surveys (December 2018-February 2019) in intervention and comparison areas

	Health centre						Health post					
	Baseline		Endline			P-value[b]	Baseline		Endline			P-value[b]
	Intervention n=74	Comparison n=81	Intervention n=68	Comparison n=74	Difference in Difference [a]		Intervention n=84	Comparison n=85	Intervention n=79	Comparison n=86	Difference in Difference [a]	
	% (95%CI)	% (95%CI)	% (95%CI)	% (95%CI)	%		% (95%CI)	% (95%CI)	% (95%CI)	% (95%CI)	%	
**Infrastructure**												
Patient toilet	99 (91-100)	96 (89-99)	97 (89-99)	97 (90-99)	-3	0.51	87 (78-93)	80(70-87)	82(72-89)	81 (72-88)	-6	0.54
Water	91 (82-96)	74 (63-82)	75 (63-84)	69 (57-79)	-11	0.36	69 (58-78)	55(44-66)	51(40-62)	52 (42-63)	-15	0.43
Fridge	77(66-85)	78(67-86)	78(66-86)	80(69-86)	-1	0.838	10(5-18)	9(10-18)	33(23-44)	40(30-50)	-8	0.027
Sterilizer	70(57-80)	63(52-73)	74(62-83)	68(59-77)	-1	0.751	10(5-18)	7(3-15)	10(5-19)	10(5-19)	-3	0.515
Electricity	59(48-70)	62(51-72)	65(52-75)	70(57-79)	-2	0.588	17(10-26)	19(12-29)	23(15-34)	25(17-36)	0	0.392
**Drugs**												
Amoxicillin [c]	97(90-99)	99(91-100)	97(89-99)	99(81-100)	0	0.975	79(68-86)	81(71-88)	73(62-82)	80(70-87)	-5	0.85
Co-trimoxazole	97 (90-99)	98 (90-99)	94 (85-98)	95 (86-98)	0	0.67	40 (30-51)	15 (9-25)	8 (3-16)	6 (2-13)	-23	0.97
Gentamicin [d]	82 (72-90)	81 (71-89)	94 (85-98)	97 (90-99)	-4	0.07	38 (28-49)	41 (31-52)	37 (27-48)	15 (9-25)	25	<0.01
Ampicillin	84 (73-91)	90 (81-95)	97 (89-99)	99 (91-100)	4	0.31						
ORS [e]	89 (80-95)	80 (70-88)	93 (83-97)	92 (83-97)	-8	0.16	76 (66-84)	58 (47-68)	92 (84-97)	85 (75-91)	-11	0.22
Zinc	12 (6-22)	7 (3-16)	16 (9-287	16 (9-27)	-5	0.21	82 (72-88)	80 (70-87)	84 (73-90)	80 (70-87)	2	0.93
Zinc-ORS[e] combined	74 (63-83)	77 (66-85)	88 (78-94)	68 (56-74)	23	0.03	10 (5-18)	5 (2-12)	14 (08-24)	9 (4-16)	0	0.59
Malaria RDT [f]	88 (78-94)	77 (66-85)	87 (76-93)	65 (53-75)	11	0.23	75 (64-83)	48 (37-59)	65 (53-74)	37 (27-48)	1	0.56
RUTF [g]	82 (72-90)	100	88 (78-94)	99 (91-100)	7		5 (2-12)	11 (06-19)	47 (36-58)	74 (64-83)	-21	<0.01
**Equipment**												
Stethoscope	100	100	99 (90-100)	100	-1		74 (63-82)	81 (71-88)	72 (61-81)	56 (45-66)	23	<0.01
MUAC [h] tape	100	99 (91-100)	99 (90-100)	99 (91-100)	-1		98 (91-99)	100	97 (90-99)	100	-1	0.95
Thermometer	100	98 (90-99)	100	99 (91-100)	-1		86 (76-92)	87 (78-93)	85 (75-91)	76 (65-84)	10	0.11
Infant scale	96 (88-99)	96 (89-99)	97 (89-99)	99 (91-100)	-2	0.49	80 (70-87)	81 (71-88)	80 (69-87)	77 (66-85)	4	0.52
Ambu face mask	95 (86-98)	94 (86-97)	96 (87-99)	96 (88-99)	-1	0.69	81 (71-88)	71 (60-79)	23 (15-34)	13 (7-22)	0	
Weighing sling	86 (76-93)	95 (87-98)	93 (83-97)	92 (83-97)	10	0.23	70 (59-79)	76 (66-84)	75 (64-83)	78 (68-86)	3	0.94
Syringes and needle	100	95 (87-98)	100	100	-5		85 (75-91)	81 (71-88)	78 (68-86)	73 (63-82)	1	0.55
Functional timer							21 (14-32)	22 (15-33)	5 (2-13)	16 (10-26)	-10	0.38
**Job aids**												
0-2 months registration book	95 (86-98)	93 (84-97)	97 (89-99)	95 (86-98)	0	0.98	92 (83-96)	88 (79-94)	94 (85-97)	86 (77-92)	4	0.53

For peer review only - http://bmjopen.bmj.com/site/about/guidelines.xhtml

2-59 months registration book	100	99 (91-100)	96 (87-99)	100	-5		95 (88-98)	89 (81-94)	95 (87-98)	94 (87-98)	-5	0.31
Chart booklet	96 (88-99)	95 (87-98)	99 (89-100)	95 (86-98)	3	0.50	87 (78-93)	87 (78-93)	94 (85-97)	90 (81-95)	4	0.77
Supervision checklist	92 (83-96)	93 (84-97)	90 (80-95)	85 (75-92)	6	0.28						
Family health card							83 (74-90)	87 (78-93)	81 (71-88)	81 (72-88)	4	0.45

[a] Difference-in-Difference: the difference in the proportion between intervention and comparison areas at endline subtracted from the difference in proportion between intervention and comparison at baseline.

[b] P-value obtained from a logistic regression model for the difference-in-difference analysis

[c] Amoxicillin 250mg, 125 mg, or 125 mg suspension

[d] Gentamicin 80mg/2ml or 20mg/2ml for the health centre and 20mg/2ml for health posts

[e] ORS: oral rehydration solution

[f] RDT: rapid diagnostic test

[g] RUTF: ready-to-use therapeutic food

[h] MUAC: middle upper arm circumference

Table 6 Median number of newborns (0-2 months) and children (2-59 months) registered in health centres and health posts in the quarter prior to survey at baseline (December 2016- February 2017) and endline surveys (December 2018-February 2019) in intervention and comparison areas

	**Health centre**						**Health post**					
	**Baseline facility survey**		**Endline facility survey**		Difference in Difference*	P-value	**Baseline facility survey**		**Endline facility survey**		Difference in Difference*	P-value[a]
	Intervention n=67	Comparison n=88	Intervention n=61	Comparison n=81			Intervention n=74	Comparison n=95	Intervention n=69	Comparison n=96		
**Age group**	Median (IQR)	Median (IQR)	Median (IQR)	Median (IQR)	Median		Median (IQR)	Median (IQR)	Median (IQR)	Median (IQR)	Median	
0-59 days	5 (8)	3(6)	9 (11)	3 (6)	4	0.08	0 (2)	0 (1)	0 (3)	0 (0)	0	>0.99
2-59 months	237 (309)	149 (171)	232 (340)	128 (119)	16	0.58	18 (37)	13 (25)	22 (33)	10 (24)	7	0.22

[a] Difference-in-Difference: the difference in the median children seen in the three months prior to the survey between intervention and comparison area health facilities at endline subtracted from the difference in the median between intervention and comparison area health facilities at baseline.
[b] P-value obtained from using quantile regression analysis.

For peer review only - http://bmjopen.bmj.com/site/about/guidelines.xhtml

Table 7 Illness, care-seeking and case-management in the two weeks prior to the survey among children 2-59 months of age reported by caregivers from the household survey at baseline (December 2016- February 2017) and endline surveys (December 2018-February 2019) in intervention and comparison areas.

	Baseline household survey		Endline household survey		Difference in Difference[a]	OR (95% CI)	Adjusted OR (95% CI) [b]
	Intervention	Comparison	Intervention	Comparison			
	% (95%CI) [n]	% (95%CI) [n]	% (95%CI) [n]	% (95%CI) [n]	%		
Illness in the last 2 weeks	6 (5-8) [n=1434]	5 (4-8) [n=1440]	13 (11-15) [n=2932]	11(9-14) [n=2707]	n/a	n/a	n/a
Among ill							
Care-seeking for any illness[c]	58 (47-68) [n=88]	49 (39-60) [n=79]	39 (32-45) [n=381]	34 (27-41) [n=308]	-4	0.62 (0.19-2.04)	0.49 (0.12-1.95)
ORS with zinc for reported diarrhoea	32 (14-56) [n=19]	25 (10-50) [n=20]	38 (25-51) [n=72]	30 (19-42) [n=47]	1	0.74 (0.04-12.26)	0.70 (0.04-13.66)
ORS with or without zinc for reported diarrhoea	53 (30-74) [n=19]	40 (18-66) [n=20]	50 (38-62) [n=72]	53 (38-68) [n=47]	-16	0.32 (0.02-4.90)	0.29 (0.02-5.33)
Antibiotic for possible pneumonia [d] symptoms	67 (16-95) [n=9]	56 (22-85) [n=9]	62 (38-81) [n=21]	69 (42-87) [n=19]	-18	0.29 (0.00-26.83)	0.15 (0.00-16.18)

[a] Difference-in-Difference: the difference in the proportion between intervention and comparison areas at endline subtracted from the difference in proportion between intervention and comparison at baseline.

[b] Adjusted for religion and distance to nearest health post. Sample size is smaller for adjusted ORs: care-seeking =127 missing data on distance; diarrhoea treatment =23 missing data on distance; antibiotics for possible pneumonia =6 missing data on distance

[c] Care-seeking from hospital, health centre, health post or private clinic

[d] Possible pneumonia cases are those children with reported cough and fast breathing or difficult breathing, but not due to a blocked nose.

For peer review only - http://bmjopen.bmj.com/site/about/guidelines.xhtml

Table 8 Morbidity, care-seeking and management among infants in the neonatal period at baseline (December 2016- February 2017) and endline surveys (December 2018-February 2019) in intervention and comparison areas.

	**Baseline household survey**		**Endline household survey**		Difference in Difference[a]	OR (95% CI)	Adjusted OR (95% CI) [b]
	Intervention	Comparison	Intervention	Comparison			
	% (95%CI) [n]	% (95%CI) [n]	% (95%CI) [n]	% (95%CI) [n]	%		
Among all							
Baby ill in the first month of life	14 (11-19) [n=331]	5 (3 -9) [n=331]	9 (7-13) [n=482]	8 (6-11) [n=397]	n/a	n/a	n/a
**Among ill**							
Prevalence of possible neonatal sepsis[c]	83 (70-91) [n=47]	78 (48-93) [n=18]	73 (59-84) [n=45]	84 (65-94) [n=32]	n/a	n/a	n/a
Care-seeking for ill baby [d]	74 (59-86) [n=47]	44 (23-69) [n=18]	51[e] (34-68) [n=43]	68[f] (49-82) [n=31]	-47	0.04 (0.00-0.49)	0.04 (0.00-0.60)
**Among possible sepsis**							
Any antibiotic	51 (34-68) [n=39]	36 (17-60) [n=14]	57 (38-73) [n=30]	68 (43-85) [n=25]	-26	0.17 (0.01-2.06)	0.19 (0.02-2.42)

[a] Difference-in-Difference: the difference in the proportion between intervention and comparison areas at endline subtracted from the difference in proportion between intervention and comparison at baseline.
[b] Adjusted for age of infant
[c] Possible neonatal sepsis: fever, unable to suckle/feed difficult/fast breathing, severe chest in-drawing, convulsions or lethargy
[d] Care-seeking outside the home
[e] Missing data for two individuals
[f] Missing data for one individual

For peer review only

For peer review only - http://bmjopen.bmj.com/site/about/guidelines.xhtml

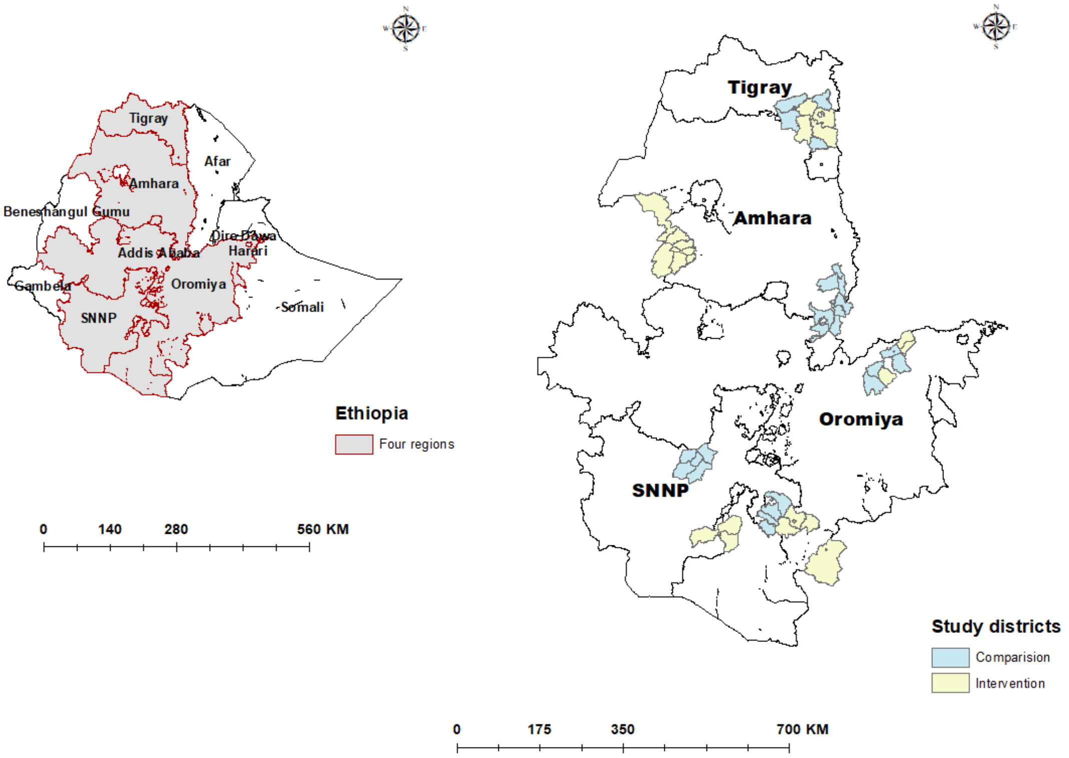
Tigray
Afar
Amhara
Beneshangul Gumu
Addis Ababa
Dire Dawa
Harari
Gambela
Oromiya
SNNP
Somali
Ethiopia
Four regions
0
140
280
560 KM
Tigray
Amhara
Oromiya
SNNP
Study districts
Comparision
Intervention
0
175
350
700 KM

review only

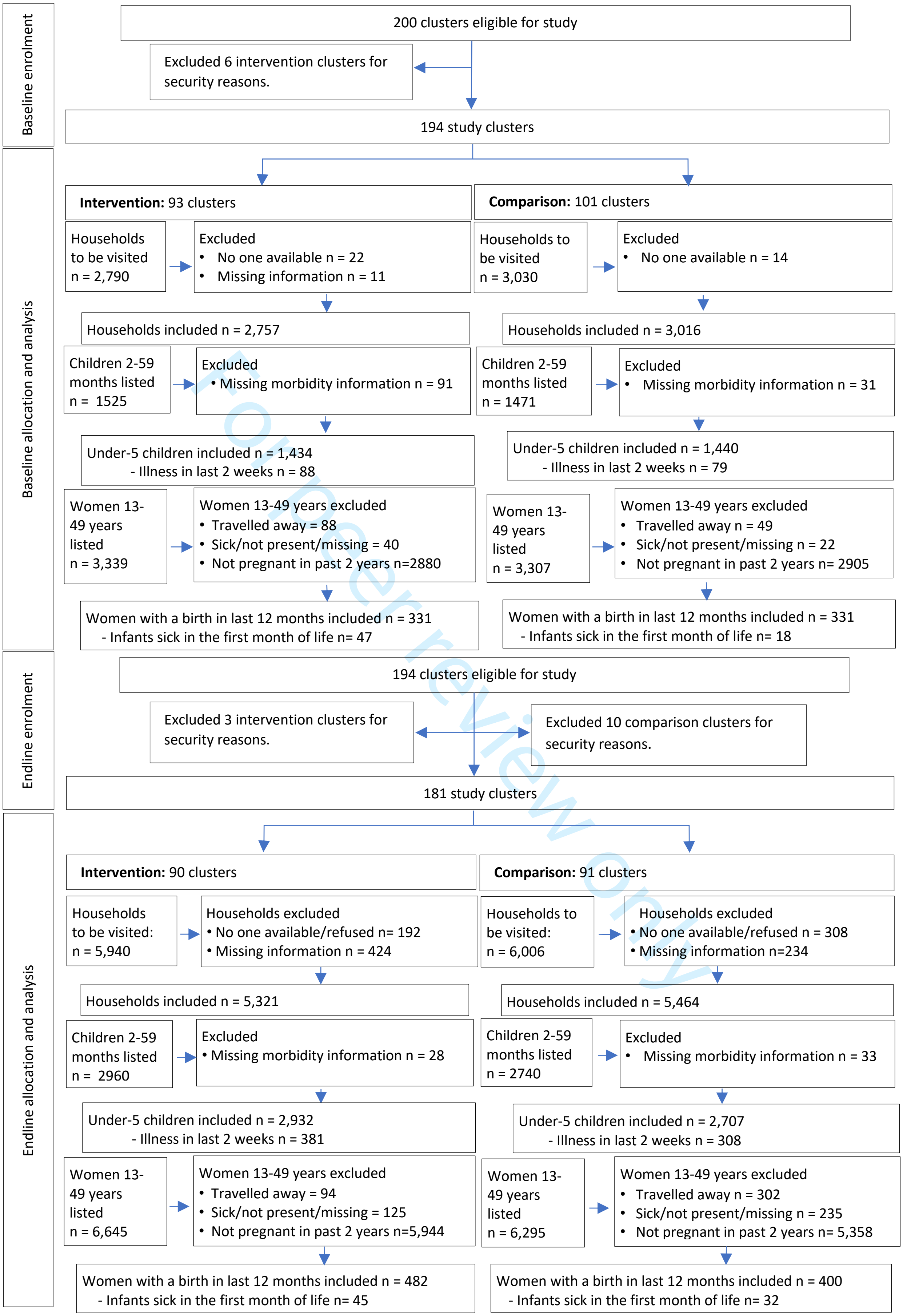

For peer review only

CONSORT 2010 checklist of information to include when reporting a randomised trial*

Section/Topic	Item No	Checklist item	Reported on page No
**Title and abstract**			
	1a	Identification as a randomised trial in the title	Not randomized
	1b	Structured summary of trial design, methods, results, and conclusions (for specific guidance see CONSORT for abstracts)	1
**Introduction**			
Background and objectives	2a	Scientific background and explanation of rationale	6-7
	2b	Specific objectives or hypotheses	7
**Methods**			
Trial design	3a	Description of trial design (such as parallel, factorial) including allocation ratio	8
	3b	Important changes to methods after trial commencement (such as eligibility criteria), with reasons	N/A
Participants	4a	Eligibility criteria for participants	9
	4b	Settings and locations where the data were collected	7-8
Interventions	5	The interventions for each group with sufficient details to allow replication, including how and when they were actually administered	7 & Table 1 (page 28)
Outcomes	6a	Completely defined pre-specified primary and secondary outcome measures, including how and when they were assessed	11
	6b	Any changes to trial outcomes after the trial commenced, with reasons	NA
Sample size	7a	How sample size was determined	9
	7b	When applicable, explanation of any interim analyses and stopping guidelines	N/A
Randomisation:			
Sequence generation	8a	Method used to generate the random allocation sequence	N/A
	8b	Type of randomisation; details of any restriction (such as blocking and block size)	N/A
Allocation concealment mechanism	9	Mechanism used to implement the random allocation sequence (such as sequentially numbered containers), describing any steps taken to conceal the sequence until interventions were assigned	N/A
Implementation	10	Who generated the random allocation sequence, who enrolled participants, and who assigned participants to interventions	7
Blinding	11a	If done, who was blinded after assignment to interventions (for example, participants, care providers, those	10 & 13

For peer review only - http://bmjopen.bmj.com/site/about/guidelines.xhtml

		assessing outcomes) and how	
	11b	If relevant, description of the similarity of interventions	N/A
Statistical methods	12a	Statistical methods used to compare groups for primary and secondary outcomes	13
	12b	Methods for additional analyses, such as subgroup analyses and adjusted analyses	13
**Results**			
Participant flow (a diagram is strongly recommended)	13a	For each group, the numbers of participants who were randomly assigned, received intended treatment, and were analysed for the primary outcome	37 Figure 1
	13b	For each group, losses and exclusions after randomisation, together with reasons	37 Figure 1
Recruitment	14a	Dates defining the periods of recruitment and follow-up	8
	14b	Why the trial ended or was stopped	8
Baseline data	15	A table showing baseline demographic and clinical characteristics for each group	29 & 30
Numbers analysed	16	For each group, number of participants (denominator) included in each analysis and whether the analysis was by original assigned groups	29 & 30
Outcomes and estimation	17a	For each primary and secondary outcome, results for each group, and the estimated effect size and its precision (such as 95% confidence interval)	31-35
	17b	For binary outcomes, presentation of both absolute and relative effect sizes is recommended	34-35
Ancillary analyses	18	Results of any other analyses performed, including subgroup analyses and adjusted analyses, distinguishing pre-specified from exploratory	31-35
Harms	19	All important harms or unintended effects in each group (for specific guidance see CONSORT for harms)	N/A
**Discussion**			
Limitations	20	Trial limitations, addressing sources of potential bias, imprecision, and, if relevant, multiplicity of analyses	19
Generalisability	21	Generalisability (external validity, applicability) of the trial findings	19
Interpretation	22	Interpretation consistent with results, balancing benefits and harms, and considering other relevant evidence	19-22
**Other information**			
Registration	23	Registration number and name of trial registry	4
Protocol	24	Where the full trial protocol can be accessed, if available	8
Funding	25	Sources of funding and other support (such as supply of drugs), role of funders	24

*We strongly recommend reading this statement in conjunction with the CONSORT 2010 Explanation and Elaboration for important clarifications on all the items. If relevant, we also recommend reading CONSORT extensions for cluster randomised trials, non-inferiority and equivalence trials, non-pharmacological treatments, herbal interventions, and pragmatic trials. Additional extensions are forthcoming: for those and for up to date references relevant to this checklist, see www.consort-statement.org.

For peer review only - http://bmjopen.bmj.com/site/about/guidelines.xhtml

BMJ Open

Does a complex intervention targeting communities, health facilities and district health managers increase the utilisation of community-based child health services? A before and after study in intervention and comparison areas of Ethiopia.

Journal:	*BMJ Open*
Manuscript ID	bmjopen-2020-040868.R1
Article Type:	Original research
Date Submitted by the Author:	23-Jul-2020
Complete List of Authors:	Berhanu, Della; London School of Hygiene and Tropical Medicine Faculty of Infectious and Tropical Diseases, Department of Disease Control; Ethiopian Public Health Institute,<br>Okwaraji, Yemisrach; London School of Hygiene & Tropical Medicine, Department of Control of Disease<br>Defar, Atkure ; Ethiopian Public Health Institute, Health Systems and Reproductive Health Research Directorate; University of Gondar College of Medicine and Health Sciences<br>Bekele, Abebe; Ethiopian Public Health Institute, Health Systems and Reproductive Health Research Directorate<br>Lemango, Ephrem Tekle; Ethiopia Ministry of Health, Maternal and Child Health Directorate<br>Medhanyie, Araya; Mekelle University College of Health Sciences<br>Wordofa, Muluemebet; Jimma University, Department of Population and Family Hea<br>Yitayal, Mezgebu ; University of Gondar College of Medicine and Health Sciences<br>W/Gebriel, Fitsum ; Hawassa University College of Medicine and Health Sciences<br>Desta, Alem ; Mekelle University College of Health Sciences<br>Gebregizabher , Fisseha; Mekelle University College of Health Sciences<br>Daka, Dawit; Jimma University, Department of Health Economics<br>Hunduma, Alemayehu; Jimma University, Department of Population and Family Health<br>Beyene, Habtamu; Hawassa University College of Medicine and Health Sciences<br>Getahun, Tigist ; University of Gondar College of Medicine and Health Sciences, College of Medicine and Health Science, Institute of Public Health<br>Getachew, Theodros ; Ethiopian Public Health Institute, Health System and Reproductive Health Research Directorate; University of Gondar College of Medicine and Health Sciences<br>Woldemariam, Amare; University of Gondar College of Medicine and Health Sciences, Department of Human Nutrition<br>Wolassa, Desta ; Ethiopian Public Health Institute,<br>Persson, Lars; London School of Hygiene and Tropical Medicine; Ethiopian Public Health Institute

	Schellenberg, Joanna; London School of Hygiene and Tropical Medicine, Disease Control
**Primary Subject Heading**:	Health services research
Secondary Subject Heading:	Health services research, Epidemiology, Infectious diseases
Keywords:	Epidemiology < INFECTIOUS DISEASES, Community child health < PAEDIATRICS, PRIMARY CARE

SCHOLARONE™
Manuscripts

I, the Submitting Author has the right to grant and does grant on behalf of all authors of the Work (as defined in the below author licence), an exclusive licence and/or a non-exclusive licence for contributions from authors who are: i) UK Crown employees; ii) where BMJ has agreed a CC-BY licence shall apply, and/or iii) in accordance with the terms applicable for US Federal Government officers or employees acting as part of their official duties; on a worldwide, perpetual, irrevocable, royalty-free basis to BMJ Publishing Group Ltd ("BMJ") its licensees and where the relevant Journal is co-owned by BMJ to the co-owners of the Journal, to publish the Work in this journal and any other BMJ products and to exploit all rights, as set out in our licence.

The Submitting Author accepts and understands that any supply made under these terms is made by BMJ to the Submitting Author unless you are acting as an employee on behalf of your employer or a postgraduate student of an affiliated institution which is paying any applicable article publishing charge ("APC") for Open Access articles. Where the Submitting Author wishes to make the Work available on an Open Access basis (and intends to pay the relevant APC), the terms of reuse of such Open Access shall be governed by a Creative Commons licence – details of these licences and which Creative Commons licence will apply to this Work are set out in our licence referred to above.

Other than as permitted in any relevant BMJ Author's Self Archiving Policies, I confirm this Work has not been accepted for publication elsewhere, is not being considered for publication elsewhere and does not duplicate material already published. I confirm all authors consent to publication of this Work and authorise the granting of this licence.

For peer review only

Does a complex intervention targeting communities, health facilities and district health managers increase the utilisation of community-based child health services? A before and after study in intervention and comparison areas of Ethiopia.

Della Berhanu[1,2*], Yemisrach B. Okwaraji[1,2], Atkure Defar[2,3], Abebe Bekele Belayneh[2], Ephrem Tekle Lemango[4], Araya Abrha Medhanyie[5], Muluemebet Abera Wordofa[6], Mezgebu Yitayal[7], Fitsum W/Gebriel[8], Alem Desta[5], Fisseha Ashebir[5,9], Dawit Wolde Daka[10], Alemayehu Hunduma[6,11], Habtamu Beyene[12,13], Tigist Getahun[3], Theodros Getachew [2,3], Amare Tariku[14] , Desta Wolassa[2], Lars Åke Persson[1,2], Joanna Armstrong Schellenberg[1]

[1] Department of Disease Control, London School of Hygiene & Tropical Medicine, London, United Kingdom.

[2] Health Systems and Reproductive Health Research Directorate, Ethiopian Public Health Institute, Addis Ababa, Ethiopia

[3] Institute of Public Health, College of Medicine and Health Sciences, University of Gondar, Gondar, Ethiopia

[4] Maternal and Child Health Directorate, Federal Ministry of Health, Addis Ababa, Ethiopia

[5] School of Public Health, College of Health Sciences, Mekelle University, Mekelle, Ethiopia

[6] Department of Population and Family Health, Institute of Family Health, Public Health Faculty, Jimma University, Jimma, Ethiopia.

[7] Department of Health Systems and Policy, Institute of Public Health, College of Medicine and Health Sciences, University of Gondar, Gondar, Ethiopia

[8] College of Medicine and Health Sciences, Hawassa University, Hawassa, Ethiopia

[9] Tigray Regional Health Bureau, Tigray, Ethiopia

[10] Department of Health Economics, Management and Policy Public Health Faculty, Jimma University, Jimma, Ethiopia

[11] Addis Ababa City Administration health Bureau, Addis Ababa, Ethiopia.

[12] College of Medicine and Health Sciences, Hawassa University, Hawassa, Ethiopia

[13] Southern Nations, Nationalities and Peoples Regional Health Bureau, Hawassa, Ethiopia

[14] Department of Human Nutrition, Institute of Public Health, College of Medicine and Health Sciences, University of Gondar, Gondar, Ethiopia

*Corresponding author: Della Berhanu; PhD, della.berhanu@lshtm.ac.uk, London School of Hygiene & Tropical Medicine, Keppel St, London WC1E 7HT

Co-authors: Yemisrach B. Okwaraji; PhD, Yemisrach.Okwaraji@lshtm.ac.uk

For peer review only - http://bmjopen.bmj.com/site/about/guidelines.xhtml

Atkure Defar; MPH, MBA, atkuredefar@gmail.com

Abebe Bekele Belayneh; MSc, abebe1277belay@gmail.com

Ephrem Tekle Lemango; MD, MA, ephremt.lemango@gmail.com

Araya Abrha Medhanyie; PhD, araya.medhanyie@gmail.com

Muluemebet Abera Wordofa; PhD, mulu_abera.ts2009@yahoo.com

Mezgebu Yitayal; PhD, mezgebuy@gmail.com

Fitsum W/Gebriel; MD, fitsedememmj@gmail.com

Alem Desta; MSc, alemdw@gmail.com

Fisseha Ashebir; MPH, fmhn2009@gmail.com

Dawit Wolde Daka; MSc, dave86520@gmail.com

Alemayehu Hunduma; MPH, hhalemayehu25@gmail.com

Habtamu Beyene; MSc, habtamu3346@yahoo.com

Tigist Getahun; MPH, tgforblessing@gmail.com

Theodros Getachew; MSc, tedi.getachew@yahoo.com

Amare Tariku; MPH, amaretariku15@yahoo.com

Desta Wolassa; MSc, desta.wolassa@lshtm.ac.uk

Lars Åke Persson; MD, PhD, Lars.Persson@lshtm.ac.uk

Joanna Armstrong Schellenberg; PhD, Joanna.Schellenberg@lshtm.ac.uk

For peer review only

ABSTRACT

Introduction Ethiopia successfully reduced mortality in children below five years of age during the past few decades, but the utilization of child health services was still low. Optimizing the Health Extension Program was a two-year intervention in 26 districts, focusing on community engagement, capacity strengthening of primary care workers, and reinforcement of district accountability of child health services. We report the intervention's effectiveness on care utilization for common childhood illnesses.

Methods We included a representative sample of 5,773 households with 2,874 under-five children at baseline (December 2016 to February 2017) and 10,785 households and 5,639 under-five children at endline surveys (December 2018 to February 2019) in intervention and comparison areas. Health facilities were also included. We assessed the effect of the intervention using difference-in-differences analyses.

Results There were 31 intervention activities; many were one-off and implemented late. In eight districts, activities were interrupted for four months. Care-seeking for any illness in the two weeks before the survey for children aged 2-59 months at baseline was 58% (95% CI 47-68) in intervention and 49% (95% CI 39-60) in comparison areas. At end-line it was 39% (95% CI 32-45) in intervention and 34% (95% CI 27-41) in comparison areas (difference-in-differences -4 percentage points, adjusted odds ratio (AOR) 0.49,95%CI 0.12-1.95). The intervention neither had an effect on care-seeking among sick neonates, nor on household participation in community engagement forums, supportive supervision of primary care workers, nor on indicators of district accountability for child health services.

Conclusion We found no evidence to suggest that the intervention increased the utilization of care for sick children. The lack of effect could partly be attributed to the short implementation period of a complex intervention and implementation interruption. Future funding schemes should take into

For peer review only - http://bmjopen.bmj.com/site/about/guidelines.xhtml

consideration that complex interventions that include behavior change may need an extended implementation period.

Trial registration: Current Controlled Trials ISRCTN12040912, http://www.isrctn.com/ISRCTN12040912

For peer review only

For peer review only - http://bmjopen.bmj.com/site/about/guidelines.xhtml

Article summary

Strengths and limitations of the study

- We conducted a rigorous effectiveness evaluation of an intervention to increase the utilization of services for sick children under-5 children using baseline (December 2016 to February 2017) and endline (December 2018 to February 2019) surveys, in intervention and comparison areas located in 56 districts across four regions of Ethiopia.
- Data were triangulated with service utilization records from health posts and health centres, which supported the household-level findings of no change in service utilization as a result of the intervention.
- Although only few of the household characteristics differed between intervention and comparison areas over time and had only a marginal influence on the analyses, it is possible that unmeasured confounders might contribute to the observed results.
- At baseline, the overall proportion of reported illness in the last two weeks was lower than anticipated, and this was higher at the endline indicating that the difference in the reported proportion of sick children could be due to differences in the interaction and the extent of the probing between data collectors and families when enquiring about childhood illnesses.
- Data collection blinded to the allocation, i.e., whether the district where they collected data was an intervention or comparison area indicating that any differential reporting of care utilization between intervention and comparison areas is, therefore, unlikely.

For peer review only - http://bmjopen.bmj.com/site/about/guidelines.xhtml

INTRODUCTION

In the period 1990-2015, Ethiopia reduced under-five mortality by 67% (1). The Ethiopian Demographic and Health Surveys (DHS) showed infant mortality rates at 77, 48, to 43 deaths per 1000 live births, in 2005, 2016, and 2019 reports, respectively (2-4). Between 2005 to 2016, neonatal mortality decreased from 39 to 29 deaths per 1000 live births, but in 2019 had stagnated at around 30. Both health system expansion and socio-economic improvements have contributed to the reductions in mortality (5).

In 2003, the Ethiopian government launched the health extension program to increase primary care services (1). Salaried female workers, known as Health Extension Workers (HEWs), were trained to provide basic community-based services. Two HEWs at the health post serve a population of approximately 5000 people. Five health posts and their referral health centre and primary hospital comprise a primary health care unit. The health extension program has 17 packages that fall under four broad areas: family health services, disease prevention and control, hygiene and environmental sanitation, and health education and communication (1). The HEWs are supported by the Women's Development Army leaders, a network of volunteer women established in 2011, who, along with other development goals, promote healthy practices in the community (6, 7).

In 2010, the integrated Community Case Management (iCCM) strategy was launched, allowing HEWs to manage common childhood illnesses in children under five years of age, including pneumonia, malaria, and diarrhoea (8). In 2013, the Community-Based Newborn Care (CBNC) was integrated into the health extension program (9). This initiative enabled HEWs to provide antibiotics for young infants with symptoms of possible serious bacterial infection when a referral was not possible.

Care-seeking for sick under-five children has remained low (10). In 2016, 46% of children with diarrhoeal diseases received oral rehydration therapy, and one-third of children with suspected pneumonia were taken to an appropriate care provider (2).

For peer review only

For peer review only - http://bmjopen.bmj.com/site/about/guidelines.xhtml

Several barriers to the utilization of services for childhood illnesses have been identified. The perceived quality of services provided by the HEWs, financial constraints, and preference for alternative care providers affected the utilization of child health services (11-13). Service uptake was associated with maternal education levels, parents' knowledge of danger signs, the type of illness, local beliefs, and the need for permission from family decision-makers to seek care (12, 14-16). Health post closure, absence of HEWs, lack of essential drugs and supplies, distance, and the quality of services also contributed to the low service utilization(12, 14, 17, 18). Insufficient supervision and lack of government accountability and ownership of child health services also constituted barriers (8).

Based on a barriers-and-facilitators study, the Ethiopian Government initiated the Optimizing the Health Extension Program (OHEP) intervention (19). OHEP was based on the following hypotheses: community engagement activities would enhance caregiver knowledge and household practices. Further, capacity strengthening would improve the availability of quality services in the CBNC and iCCM programs, and promotion of district-level ownership and accountability would integrate these services into the district level planning and budgeting. These different components together would hypothetically lead to increased utilization of CBNC and iCCM services.

This study aimed to assess the extent to which the OHEP intervention increased care-seeking for children under the age of five years, by comparing changes over time in intervention and comparison areas. Secondary outcomes included treatment of sick children with diarrhoea, suspected pneumonia, or neonatal sepsis.

METHODS

Study setting

The Ethiopian Government initiated the OHEP intervention in 26 districts of Amhara, Southern Nation, Nationalities and Peoples, Oromia, and Tigray regions with an approximate population of 3.5 million

For peer review only - http://bmjopen.bmj.com/site/about/guidelines.xhtml

(Figure 1). Intervention districts were selected by the government of Ethiopia and implementing partners for having both a relatively low utilization of primary child health services and the availability and ability of partners to support implementation. The implementers were four Non-Governmental Organizations (PATH, UNICEF, Save the Children, and Last 10 Kilometres/John Snow Inc.). The intervention started in 2016 and lasted for a duration of 2.5 years and had three components: 1) community engagement, 2) primary care level capacity building, and 3) ownership and accountability of child health services at the district level. The intervention activities under these components, along with the underlying assumptions, intermediate indicators, and outcomes are detailed in Table 1.

Figure 1 Map of Ethiopia showing all regions (left) and the intervention and comparison districts within the four study regions (right).

Table 1 Optimizing the Health Extension Program intervention implemented in 26 districts of Ethiopia, the assumptions and the expected intermediate indicators and outcomes.

Study design

The protocol for the evaluation of the OHEP implementation has been published (20). This study was based on a plausibility design with 26 intervention and 26 comparison districts (woredas) in four regions of Ethiopia. The baseline survey was conducted from December 2016 to February 2017 and the endline survey from December 2018 to February 2019 (Figure 1). The surveys were conducted by the London School of Hygiene and Tropical Medicine and Ethiopian Public Health Institute along with representatives from Gondar, Jimma, Mekelle and Hawassa Universities.

Sample

Intervention districts had a low coverage of maternal, newborn, and child health indicators. Comparison districts were selected by the Regional Health Bureaus to be similar to the size of the population, the

For peer review only - http://bmjopen.bmj.com/site/about/guidelines.xhtml

burden of diseases, number of primary health care units, health service coverage, length of iCCM and CBNC service delivery, and absence of partners implementing other demand generation activities.

We used a two-stage stratified cluster sampling to select a representative sample of households within intervention and comparison areas. In the first stage, a list of all enumeration areas of the study districts was obtained based on the 2007 Ethiopian Housing and Population Census. Two hundred enumeration areas (clusters) were selected with probability proportional to the size of the district. Each cluster served as the primary sampling unit. Within clusters, households were selected by systematic random sampling. The Women's Development Army leaders, HEWs, health posts, health centres with staff, and woreda health offices serving the selected clusters were also surveyed.

The sample size was calculated to measure changes with adequate power in a fixed number of percentage points of key indicators between intervention and comparison areas at baseline and endline. Based on the Ethiopian DHS data, a cross-sectional survey of 3000 households in 100 intervention and 100 comparison clusters was expected to find 1747 children under the age of five years in each arm (21). A Tanzanian childhood study found that 50% of under-fives had an illness in the two weeks before the survey (22). The current research assumed more conservatively 30% of children 2-59 months to have had any illness during the two weeks before the interview. This sample size of 3000 households per group with 90% completeness and a design effect of 1.3 would give 80% power to detect differences of 10-20 percentage points across a range of child health indicators (5% significance level). The baseline survey found fewer than the expected number of sick children in the two weeks before the survey. As a result, the household sample size for the endline survey was doubled. We used the baseline list of households to select 66 households randomly in each enumeration area.

For peer review only - http://bmjopen.bmj.com/site/about/guidelines.xhtml

Measurements

For every selected household, we interviewed the household head, listed residents, and collected sociodemographic characteristics. The interviews included caregivers of children aged 2-59 months to assess their knowledge of childhood illnesses and care-seeking for sick under-five children in the two weeks before the survey. Further, women of reproductive age (13 to 49 years) were interviewed to identify births in the 12 months before the survey, with additional questions on care-seeking for illness in the neonatal period. Up to three visits were made to each participant to ensure the study reaches its target sample size.

The health facility questionnaires assessed the infrastructure, equipment, supplies, and staff available on the day of the survey. Also, data were collected from facility registers on services provided to sick children at health posts and health centres in the three months preceding the survey. The health centre staff, HEWs, and Women's Development Army leader modules covered their background, knowledge, training in the last twelve months, supervision in the previous six months and the services they provided in the last three months. Interviewers collected information at the district health office on demography and characteristics that might affect services for under-five children.

The questions and content of each survey module were based on existing large-scale survey tools and the authors' previous evaluation of the iCCM and CBNC programs (22, 23). All questionnaires were translated into three local languages (Amharic, Oromifa, and Tigrigna), pre-tested and revised. Data collectors and supervisors were trained over ten days, including field training before the start of data collection. They were not provided information on whether a district was an intervention or comparison area.

Data were collected on personal digital assistants (Companion Touch 8), and tablets (Toshiba and Hewlett Packard) programmed with CSPro 6.3 at baseline and CSPro 7.1 at the endline. Data collectors sent encrypted data from the field to the password-protected server at the Ethiopian Public Health Institute.

For peer review only - http://bmjopen.bmj.com/site/about/guidelines.xhtml

Data managers conducted quality checks and feedback to field teams. Data were cleaned, checked for errors, including consistency and completeness. Since OHEP was a community and health system level intervention, no data monitoring committee was not deemed necessary.

Outcomes

The primary outcome was the proportion of children 2-59 months old who were reported to have had any illness in the two weeks before the survey for whom advice or treatment was sought from an appropriate provider (health post, health centre, hospital, and private clinic).

Secondary outcomes included: 1) the proportion of sick 2-59 months old children who were reported to have received appropriate treatment for diarrhoea (oral rehydration solution (ORS) with or without zinc tablets) and possible pneumonia (antibiotics), 2) the proportion of infants born in the 12 months before the survey who were reported to have had any illness in the first 28 days of life for whom advice or treatment was sought from an appropriate provider, and 3) the proportion of infants born in the 12 months before the survey who received adequate treatment for suspected neonatal sepsis (antibiotics). Since malaria was not a common illness in the study areas at the time of the surveys, the assessment of appropriate treatment for this illness was excluded. Additionally, the registers for children aged 0-59 days and 2-59 months old were reviewed to assess the median number of children seeking care at health centres and health posts in the three months before the surveys.

We also evaluated intermediate indicators that included, at the community level, the proportion of caregivers that knew signs of illness in children and the proportion that cited appropriate action to be taken for a sick child under five years of age. We also assessed the proportion of caregivers that reported receiving health messages on common childhood illnesses and those attending community meetings to discuss maternal, newborn, and child health issues. At the health system level, we assessed the proportion of HEWs that had received training and the proportion that had attended performance

For peer review only - http://bmjopen.bmj.com/site/about/guidelines.xhtml

review and clinical mentoring meetings. We also evaluated the proportion of health centres and health posts that had received supervision and the proportion that had the necessary equipment, supplies, and drugs for the provision of child health services. District-level ownership and accountability for child health programs were reflected in the proportion of districts that had iCCM and CBNC scorecards. These cards were program management tools for setting targets and monitoring performance. Information was also collected on the average number of ambulances available in districts to transport sick under-five children and whether there were standardized hours of operation for health posts.

Analyses

Descriptive analysis of baseline and endline characteristics in intervention and comparison areas was conducted at the household, caregiver, child, health facility, and district levels. Categorical variables were summarized using percentages with 95% confidence intervals. We used means, with standard errors, or medians, with interquartile ranges, to summarise continuous variables.

At the district level, the demographic and health system level characteristics were examined. For households, the characteristics of mothers or caregivers of children aged 2-59 months, and women who had a delivery in the 12 months before the survey were assessed. Distribution of age, religion, education, self-reported distance to the nearest health post, and socioeconomic status was analysed in intervention and comparison areas at baseline and endline surveys. Similar assessments were done for the distribution of age and sex among children 2-59 months of age and infants born during the 12 months before the survey. Household socioeconomic status was captured by asset ownership, access to utilities, and household characteristics. These were aggregated into a single wealth index score using principal component analysis (24). The household aggregated scores were grouped into wealth quintiles, where quintile 1 represented the poorest fifth of the households, and quintile 5 represented the least poor fifth. A linear or logistic regression model was fitted, depending on the variable type, to assess if

For peer review only - http://bmjopen.bmj.com/site/about/guidelines.xhtml

there were any differences between intervention and comparison areas that changed over time. A variable was considered a potential confounder if the differences between intervention and comparison areas showed a statistically significant (p-value <0.05) change over time.

Caregivers' knowledge, practice, and community participation relating to child health were assessed. Further, the health system level factors associated with child health services, including training and supervision of health workers providing under-five services, and the observed availability of infrastructure, equipment, supplies, and drugs for the treatment of childhood illnesses at health posts and health centres were assessed.

Using data from facility registers, we also compared the median number of young infants and children 2-59 months of age who received care in the three months before the survey in intervention and comparison areas at baseline and endline. We analysed for differences between intervention and comparison areas over time using quantile regression analysis.

Difference-in-differences analyses were used to estimate the effect of the OHEP intervention on care-seeking for sick under-five children. Binary outcome indicators were used to capture whether a sick child had sought care or received treatment. The key independent variable for the outcomes of this study was whether the child lived in the OHEP intervention or comparison area. A model was then created that included an interaction term for the timing of the survey (baseline or endline) and the survey area (OHEP intervention or comparison area). This model allowed for the calculation of the odds of care-seeking or treatment for under-five children in intervention areas as compared to comparison areas, accounting for any differences between baseline and endline survey areas, with adjustment for the cluster sampling and identified confounding factors. The Stata 13 (Stata Corporation, College Station, Texas) svy commands were used to adjust for clustering. The assessment used a blinded analysis. The

For peer review only - http://bmjopen.bmj.com/site/about/guidelines.xhtml

code identifying the intervention and comparison areas were revealed after the analysis and interpretations were completed.

Patient and public involvement

Patient and/or the public were not involved in the design or conduct, or reporting or dissemination plans of this research.

RESULTS

The OHEP intervention started in some districts in 2016 and was fully operational from the start of 2017 until October 2018. A process evaluation of the implementation will be reported elsewhere (Okwaraji et al., submitted for peer review) and includes an assessment of implementation fidelity as well as qualitative results on successes and challenges of the implementation. Briefly, a majority of the activities were one-off, assuming that health extension workers would catalyse these activities in the communities. The fidelity of the 31 activities varied by district and over time. Many activities were delayed to the last year. In eight of the districts, implementation activities were interrupted 4 months for administrative reasons. Civil unrest in some of the districts had an impact on health services.

Out of the 200 clusters eligible, six intervention clusters were excluded at baseline due to civil unrest and, therefore, not included in the endline survey (Figure 2). At endline, a further three intervention clusters and ten comparison clusters were excluded due to civil unrest.

Figure 2 Study flow diagram of intervention and comparison categorised by baseline and endline data collection period.

District-level data were not available from five intervention and one comparison districts at baseline, while at endline data were missing from four intervention and two comparison districts (S1 Table). The demographic and health systems characteristics of intervention and comparison districts at baseline and

For peer review only - http://bmjopen.bmj.com/site/about/guidelines.xhtml

endline surveys were similar. The study districts had an average population size of 130,000 inhabitants, with 23% being women 15-49 years, 20% being children below the age of five years. The average household size was 4.6 persons. One-third of the districts had a hospital. There were, on average, five health centres per district and five health posts under each health centre. At the time of the baseline survey, there were 1-2 ambulances available on average in the district for transporting sick children, while this had increased to 2-3 at the time of the endline survey. There were also some increases in staffing; health officers at the health centres increased from 2 to 2.5, midwives at health centres from 2.5 to more than 3, and the number of HEWs increased from an average slightly above 2 per health post to almost 3. These characteristics were similar across intervention and comparison districts. At the time of the baseline survey, CBNC and iCCM indicators were included in the scorecards of 79% of intervention and over 90% of comparison districts. At the endline, over 90% of all districts had iCCM and CBNC indicators in their scorecards.

The distribution of study participants by age, education, and socio-economic status across intervention and comparison areas showed no evidence of a change over time. There was some evidence that the proportion of Orthodox Christians changed over time, as did the reported distance to the nearest health post. Both were included as confounders in the analysis of the primary outcome. The distribution of sex and age of under-five children remained similar in the study areas over time (Table 2). The characteristics of women who had delivered in the 12 months before the survey showed no evidence of change over time. Young infant age distribution changed, which was included as a potential confounder in the analyses (Table 3).

Table 2 Characteristics of mothers or caregivers of children aged 2-59 months and of their children at baseline (December 2016- February 2017) and endline surveys (December 2018-February 2019) in intervention and comparison areas.

For peer review only - http://bmjopen.bmj.com/site/about/guidelines.xhtml
For peer review only

Table 3 Characteristics of mothers and their children born in the 12 months prior to the survey at baseline (December 2016- February 2017) and endline surveys (December 2018-February 2019) in intervention and comparison areas.

There was no association between the intervention and child health messages the caregivers had received or attendance at community health-related meetings (S2 Table). Between baseline and endline, caregivers' unprompted knowledge on sick newborn danger signs, such as limited or no movement and skin pustules, showed some increase in intervention areas as compared to comparison areas over time (S3 Table). At baseline and endline, over four-fifths of the caregivers in intervention as well as comparison areas said they would take their child to the health centre for a range of childhood illnesses, while approximately one-fifth said they would go to a health post (S4 Table).

Two or more HEWs were available in most health posts without association to the intervention (p=0.55 in difference-in-differences analysis) (S5 Table). Most health posts were open five days a week without any association with the intervention (p=0.99 in difference-in-differences analysis). The proportion of health posts that posted their operational days and hours decreased in both areas over time.

The proportion of HEWs trained in iCCM and CBNC remained similar at baseline and endline in intervention and comparison areas (Table 4). Maternal, newborn, and child health orientation for Women's Development Army leaders decreased slightly in intervention areas and had a minor increase in comparison areas (p=0.34 in difference-in-differences analysis).

Table 4 Child health training for health extension workers and Women's Development Army leaders at baseline (December 2016- February 2017) and endline surveys (December 2018-February 2019) in intervention and comparison areas

At baseline, approximately two-thirds of health centre staff in the intervention and comparison areas reported receiving a supervisory visit in the last three months; this remained similar at endline (S6 Table). Over half of all the surveyed HEWs at baseline and endline reported receiving a supervisory visit

For peer review only - http://bmjopen.bmj.com/site/about/guidelines.xhtml

in the last month. Approximately half in both areas and at both time points reported attending a performance review and clinical mentoring meeting in the six months before the survey. One in six Women's Development Army leaders reported meeting with HEWs and other leaders in the three months prior to the survey in intervention and comparison areas at baseline and remained similar over time.

Almost all health centres and most health posts had patient toilets (Table 5). Water availability on the day of the survey decreased over time in intervention area health centres and health posts, and at endline about three-quarters of health centres and half of the health posts in both study areas had water. Electricity on the day of the survey was available in approximately two-thirds of the health centres and one-fifth of the surveyed health posts at baseline and endline.

Table 5: Observed availability of infrastructure, equipment, supplies and drugs for treatment of childhood illness at health posts and health centres at baseline (December 2016- February 2017) and endline surveys (December 2018-February 2019) in intervention and comparison areas

At baseline and endline, antibiotics needed to treat sick under-five children were available in most health centres (Table 5). At health posts, over three-quarters had amoxicillin. However, gentamicin was not available in over half of the intervention and comparison area health posts at baseline and endline. ORS and zinc were available in almost all health centres at both time points. Zinc was also available in most health posts at both time points. ORS availability increased over time in both intervention and comparison areas. Almost all health centres had job aids and equipment needed for managing sick under-five children. Health posts lacked some equipment in both study areas at baseline and endline, while most had the necessary job aids at both time points.

The median number of sick young infants registered in intervention area health centres in the quarter before the survey at baseline was five and increased to nine at the endline, while the median remained at three for comparison areas health centres (p=0.08) (Table 6). Intervention area health centres at

For peer review only - http://bmjopen.bmj.com/site/about/guidelines.xhtml

baseline and endline had registered a similar median number of sick 2-59 months old children (237 vs. 232), while comparison area health centres registered fewer (149 vs. 128) median number of cases (p=0.58). The median number of sick young infants seen at intervention and comparison area health posts for the quarter before the survey was zero at both baseline and endline. The median number of children 2-59 months seen increased from 18 to 22 in intervention areas and decreased from 13 to 10 in comparison areas (p=0.22).

Table 6 Median number of newborns (0-2 months) and children (2-59 months) registered in health centres and health posts in the quarter prior to survey at baseline (December 2016- February 2017) and endline surveys (December 2018-February 2019) in intervention and comparison areas.

At baseline, 6% of intervention and 5% of comparison area caregivers reported that their 2-59 months old children had been sick in the two weeks before the survey (Table 7). At endline, reported illness had increased (13% and 11%). Care-seeking for any illness at baseline in intervention areas was 58% and 49% in comparison areas, while at endline it was 39% in intervention areas and 34% in comparison areas (difference-in-differences -4 percentage points, adjusted odds ratio (AOR)=0.49 (95% CI: 0.12-1.95)). The use of ORS with zinc for diarrhoea was 32% in the intervention areas and 25% in comparison areas at baseline, while at endline it was to 38% in intervention areas and 30% in comparison areas (difference-in-differences +1, AOR =0.70 (95% CI: 0.04-13.66)). The use of ORS with or without zinc was higher in intervention than in comparison areas (53% vs. 40%) at baseline, while at endline it was similar (50% vs. 53%) in both areas (difference-in-differences -16, AOR = 0.29 (95% CI: 0.02-5.33)). Antibiotic treatment for reported signs and symptoms of possible pneumonia in the intervention areas was higher than in comparison areas at baseline (67% vs. 56%). At endline, it was 62% in intervention and 69% in comparison areas (difference-in-differences -18, AOR =0.15 (95% CI:0.00-16.18)).

Table 7 Illness, care-seeking and case-management in the two weeks prior to the survey among children 2-59 months of age reported by caregivers from the household survey at baseline (December 2016-

For peer review only - http://bmjopen.bmj.com/site/about/guidelines.xhtml

February 2017) and endline surveys (December 2018-February 2019) in intervention and comparison areas.

Among those that reported illness in the neonatal period, over 70% reported signs and symptoms of possible sepsis (Table 8). At baseline care-seeking for any neonatal illness was higher in intervention as compared to comparison areas (74% vs. 44%), while at endline it was lower (51% vs. 68%) in the intervention than in comparison areas (difference-in-differences -47, AOR =0.04 (95% CI: 0.00-0.60)). Antibiotic treatment among those with possible sepsis at baseline was 51% in the intervention and 36% in comparison areas and. At endline, it was 57% in the intervention and 68% in comparison areas (difference-in-differences -26, AOR =0.19 (95% CI: 0.02-2.42)).

Table 8 Morbidity, care-seeking and management among infants in the neonatal period at baseline (December 2016- February 2017) and endline surveys (December 2018-February 2019) in intervention and comparison areas.

DISCUSSION

This study found that the Optimizing the Health Extension Program intervention neither had any effect on care-seeking for any illness nor on treatment for diarrhoea or possible pneumonia in children 2-59 months of age. Neither did we find an evidence of an effect on care-seeking for neonatal illness nor on the treatment of possible serious bacterial neonatal infection. These findings were based on household surveys and were supported by results from register reviews at health posts and health centres that showed a low level of service utilization for sick under-five children at baseline and endline surveys. The intervention did not affect caregivers' participation in community engagement activities. No changes were observed in facility preparedness in health centres and health posts that could be related to the intervention. The health system characteristics at the district level showed small changes, which were not associated with the intervention.

For peer review only - http://bmjopen.bmj.com/site/about/guidelines.xhtml

The OHEP intervention took place in selected districts of four regions of Ethiopia. Study population characteristics in intervention and comparison districts were broadly similar. A few of the household characteristics differed between intervention and comparison areas but had a marginal influence on the analyses. It is, however, possible that unmeasured confounders might contribute to the observed results. Given that child morbidity has a seasonal variation, both surveys were done during the same months of the year. At baseline, the overall proportion of reported childhood illness in the last two weeks was lower than anticipated, and this was higher at the endline. The difference in the reported proportion of sick children could be due to differences in the interaction and the extent of the probing between data collectors and families when enquiring about childhood illnesses. Field interviewers were, however, blinded to the allocation, i.e., whether the district where they collected data was an intervention or comparison area. Any differential reporting of care utilization between intervention and comparison areas is, therefore, unlikely. Service utilisation records from health posts and health centres supported the household-level findings of no change in service utilization as a result of the intervention.

The three components of the OHEP intervention were based on an analysis of barriers to child health services utilization in Ethiopia (19). As one of the three components, OHEP included several strategies to engage community members, which have been reported to be effective in Ethiopia and elsewhere (25-27). Under this intervention, OHEP engaged male agricultural extension workers to reach male partners to address the identified barrier of mothers needing permission from family decision-makers to seek care. The health post open house introduced the newborn and child health services available free of charge to the community. This activity addressed the lack of knowledge of the health post services as well as the perceptions of poor-quality care and costs for seeking care provided by the HEWs. Engaging schoolteachers also aimed to increase the awareness of health post services among parents, via their children. Parents' lack of knowledge of danger signs, preference for alternative care and local beliefs

For peer review only - http://bmjopen.bmj.com/site/about/guidelines.xhtml

that hindered care seeking for ill children were tackled by involving religious and traditional leaders and communicating behaviour change thorough radio spots and dramas, educational films, family health guide (a low literacy pictorial guide to promote maternal, newborn and child health), posters and brochures. This study found that the OHEP intervention neither improved the reach of health messages on treatment for childhood illnesses to caregivers, nor did it influence their community engagement related to maternal, newborn, and child health issues. Most caregivers also indicated that they would rather take their sick child to a health centre than to the health post.

The second component of the OHEP intervention aimed to build capacity through training, supportive supervision, and performance review and clinical mentoring to address the identified barrier of poor quality of services provided by HEWs. No changes were observed in the iCCM and CBNC training of HEWs that could be related to the intervention. Training alone may not be sufficient to improve health care provider's performance (28). Studies conducted in Ethiopia on supportive supervision and performance review and clinical mentorship meetings have also shown that these can be effective means of improving services provided by HEWs (29). The OHEP intervention had no effect on the supervision and the performance review and clinical mentoring meetings provided for HEWs. The quality of iCCM services measured at baseline was low, and it is unlikely to have been improved by the training, supervision, and infrequent clinical mentoring supported by OHEP (18).

It is essential to ensure that the actors who are involved in the provision of childhood services are accountable in the development, financing, implementation, and monitoring of the programs (30). The third component of OHEP, district level ownership and accountability, aimed to address the barrier of health post closure and absence of HEWs by working with the district health offices to standardize and display the health post operational days and hours. Very few health posts displayed this information at the time of the endline survey. Lack of essential drugs and supplies were also identified as barriers and OHEP advocated at the district level to ensure that budget was allocated for their purchase. We found

For peer review only - http://bmjopen.bmj.com/site/about/guidelines.xhtml

that almost all health centres had ORS, amoxicillin, and gentamicin, and nearly all health posts had ORS. Some health posts did not have amoxicillin, and most did not have gentamicin. Compared to another recent Ethiopian study, amoxicillin availability was higher , while the availability of gentamycin was similar (8). To address the problem of distance, implementers also advocated that district ambulances be used to transport sick children. Although more ambulances were available at the endline, the increase was not linked to the intervention.

In the current study, approximately half of the ill children 2-59 months of age sought care at baseline, and this decreased to one-third at the endline. Other studies have reported similar and even lower levels of iCCM service uptake in Ethiopia (2, 14, 31). Our baseline result for care-seeking was higher than expected, while the endline proportion follows the gradually increasing trend seen in the DHS surveys (2, 4, 21). Treatment for diarrhoea estimated in this study seemed to follow a plausible upward trend seen across the DHS. The estimated antibiotic treatment for possible pneumonia, which was less than 10% in 2005 and 2011 DHS, was two-thirds in our study. It should be noted that household surveys may not provide a reliable estimate of treatment for possible pneumonia (32, 33). It is important to consider the context in which OHEP was implemented to understand the outcomes in this evaluation. One plausible explanation for the lack of observed effect may be the relatively short duration of the intervention (34). In comparison with single or multi-component intervention, complex social and health systems interventions, that have many sequential activities, should not be expected to result in rapid effects on care utilization (35). In some districts, several intervention activities were only fully implemented in the second year, i.e., late in the project period. It is also possible that the interaction between different components in a complex intervention within a particular setting might introduce unpredictable effects (36). Some intervention activities, such as the health post open house, were also one-off efforts that were unlikely to have a sustained effect on service utilization. The assumptions under which the OHEP intervention was expected to succeed, for example, community influencers

For peer review only - http://bmjopen.bmj.com/site/about/guidelines.xhtml

becoming change agents to promote child health services, might not have been met. The lack of drugs, particularly for CBNC services, also indicates that the assumption that the public sector and supply chain partners would ensure drug availability was not fully met. In some districts, implementation was also interrupted for several months due to challenges of reaching administrative agreement between implementing and sub-contracting partners. Implementation in other districts was also interrupted due to civil unrest, which made it unsafe for project staff to conduct intervention activities. However, it is important to note that civil unrest was present in a similar number of intervention and comparison clusters. Delays were also caused by difficulties in procuring and supplying behaviour change communication tools. Lastly, the OHEP logic framework developed from the analysis of barriers to child health service utilization in Ethiopia could have benefited from including a behavioural change theory given that the main outcome, care seeking, required a change in behaviour.

Future interventions should consider other strategies with evidence of improving child health outcomes. Such strategies might include proactive case detection of ill children by Women's Development Army leaders and HEWs through regular door-to-door home visits (37). In addition to engaging communities, supporting HEWs through the Women's Development Army leaders can also improve child health outcomes (38). A linked study has shown that at while two-thirds of Women's Development Army leaders had monthly contact with HEWs, their overall knowledge on maternal, newborn and child health was low (39). While OHEP aimed to improve the competency of Women's Development Army leaders, this aspect of the intervention was poorly implemented (Okwaraji et al., submitted for peer review). Improving the Women's Development Army leaders knowledge of danger signs so they can convey health messages regularly to their peers as well as identify and refer ill children to health posts could improve services for under-five children (40).

For peer review only - http://bmjopen.bmj.com/site/about/guidelines.xhtml

CONCLUSION

This evaluation in four regions of Ethiopia showed that the OHEP intervention did not have an effect on care-seeking for sick under-five children. The lack of effect could be attributed to the relatively short period of OHEP implementation, the nature and unmet assumptions of the intervention, and implementation interruption. Future funding schemes need to take into consideration that complex interventions with multiple components, including behaviour change, need a more extended implementation period to measure the effectiveness of the program. This evaluation is linked to an ongoing process evaluation as well as in-depth sub-studies that address the Women's Development Army leaders' promotion of maternal and child health, quality of care provided by HEWs, equity, and geographic distribution of service utilization that can also offer further explanations to the observed lack of effect (18, 39, 41, 42). Given the overall low care seeking for childhood illnesses in this study continued efforts are needed to strengthen the primary care services for under-five children.

Authors' contributions

The study was conceived by JAS and ETL with inputs from DB, YO, ADef, ABB, AAM, MY, MA, FWB, ADes, FA, DWD, HB, AT, and LP. Contributions to training of data collectors, piloting and supervision during data collection were done by DB, YO, ADef, LP, ADes, FA, DWD, HB, AT, TG, TG, AH. DW was the data manager. JS, LP, YO, DB and AD analysed and interpreted the data. DB prepared the first draft of the manuscript with contributions from JS, LP, YO and AD. All authors read and commented on the manuscript and approved the final version.

For peer review only - http://bmjopen.bmj.com/site/about/guidelines.xhtml

Acknowledgements

The authors would like to thank Lindsay Mangham-Jefferies for drafting the initial protocol and questionnaires for the surveys. The authors would also like to thank the field teams that were involved in the data collection as well as the government official that facilitated the administration of the surveys. They also thank the study participants who agreed to generously give their time to participate in study.

Ethical considerations

Ethical approvals were obtained from the Ethiopian Public Health Institute (Ethics Ref 613/52) and the London School of Hygiene & Tropical Medicine (Ethics Ref 16117). Information sheets translated into the three local languages were read out to study participants to obtain their written informed consent.

Data availability statement

Request for data can be made to Della Berhanu (della.berhanu@lshtm.ac.uk). A data sharing committee has been established. All requests will be reviewed by this committee and if granted, data will be shared without any identifiers.

Funding

This project was funded by Bill & Melinda Gates Foundation (OPP1132551). The funder had no role in the study design, collection, management, analysis, or interpretation of data.

Competing interests

The authors declare that they have no competing interests.

For peer review only

For peer review only - http://bmjopen.bmj.com/site/about/guidelines.xhtml

REFERENCES

1. Assefa Y, Gelaw YA, Hill PS, Taye BW, Van Damme W. Community health extension program of Ethiopia, 2003-2018: successes and challenges toward universal coverage for primary healthcare services. Global Health. 2019;15(1):24.
2. Central Statistical Agency (CSA) and ICF International. Ethiopia Demogrpahic and Health Survey 2016. Addis Ababa, Ethiopia and Rockville, Maryland, USA. CSA and ICF. 2016.
3. Ethiopian Public Health Institute (EPHI) and ICF International. Ethiopian Demogrpahic and Health Survey 2019. Addis Ababa, Ethioia and Rockville, Marlyand, USA. EPHI and ICF. 2019.
4. Central Statistical Agency (CSA) and ICF International. Ethiopian Demogrpahic and Health Survey 2005. Addis Ababa, Ethiopia and Rockville, Maryland, USA. CSA and ICF. 2006.
5. Ruducha J, Mann C, Singh NS, Gemebo TD, Tessema NS, Baschieri A, et al. How Ethiopia achieved Millennium Development Goal 4 through multisectoral interventions: a Countdown to 2015 case study. Lancet Glob Health. 2017;5(11):e1142-e51.
6. Yitbarek K, Abraham G, Morankar S. Contribution of women's development army to maternal and child health in Ethiopia: a systematic review of evidence. BMJ Open. 2019;9(5):e025937.
7. Damtew ZA, Karim AM, Chekagn CT, Fesseha Zemichael N, Yihun B, Willey BA, et al. Correlates of the Women's Development Army strategy implementation strength with household reproductive, maternal, newborn and child healthcare practices: a cross-sectional study in four regions of Ethiopia. BMC Pregnancy Childbirth. 2018;18(Suppl 1):373.
8. Hailemariam S, Gebeyehu Y, Loha E, Johansson KA, Lindtjorn B. Inadequate management of pneumonia among children in South Ethiopia: findings from descriptive study. BMC Health Serv Res. 2019;19(1):426.
9. Mathewos B, Owen H, Sitrin D, Cousens S, Degefie T, Wall S, et al. Community-Based Interventions for Newborns in Ethiopia (COMBINE): Cost-effectiveness analysis. Health Policy Plan. 2017;32(suppl_1):i21-i32.
10. Amouzou A, Hazel E, Shaw B, Miller NP, Tafesse M, Mekonnen Y, et al. Effects of the integrated Community Case Management of Childhood Illness Strategy on Child Mortality in Ethiopia: A Cluster Randomized Trial. Am J Trop Med Hyg. 2016;94(3):596-604.
11. Tefera W, Tesfaye H, Bekele A, Kayessa E, Waltensperger KZ, Marsh DR. Factors influencing the low utilization of curative child health services in Shebedino District, Sidama Zone, Ethiopia. Ethiop Med J. 2014;52 Suppl 3:109-17.
12. Shaw B, Amouzou A, Miller NP, Tafesse M, Bryce J, Surkan PJ. Access to integrated community case management of childhood illnesses services in rural Ethiopia: a qualitative study of the perspectives and experiences of caregivers. Health Policy Plan. 2016;31(5):656-66.
13. King R, Jackson R, Dietsch E, Hailemariam A. Barriers and facilitators to accessing skilled birth attendants in Afar region, Ethiopia. Midwifery. 2015;31(5):540-6.
14. Shaw B, Amouzou A, Miller NP, Tsui AO, Bryce J, Tafesse M, et al. Determinants of Utilization of Health Extension Workers in the Context of Scale-Up of Integrated Community Case Management of Childhood Illnesses in Ethiopia. Am J Trop Med Hyg. 2015;93(3):636-47.
15. Nigatu SG, Worku AG, Dadi AF. Level of mother's knowledge about neonatal danger signs and associated factors in North West of Ethiopia: a community based study. BMC Res Notes. 2015;8:309.
16. Degefie T, Amare Y, Mulligan B. Local understandings of care during delivery and postnatal period to inform home based package of newborn care interventions in rural Ethiopia: a qualitative study. BMC Int Health Hum Rights. 2014;14:17.
17. Tilahun H, Fekadu B, Abdisa H, Canavan M, Linnander E, Bradley EH, et al. Ethiopia's health extension workers use of work time on duty: time and motion study. Health Policy Plan. 2017;32(3):320-8.

For peer review only - http://bmjopen.bmj.com/site/about/guidelines.xhtml

18. Getachew T, Mekonnen S, Yitayal M, Persson LA, Berhanu D. Health Extension Workers' diagnostic accuracy for common childhood illnesses in four regions of Ethiopia: a cross-sectional study. Acta Paediatr. 2019.
19. UNICEF and PATH. Literature Review on Barriers to Utilization of Health Extension Services: Draft Report 2016.
20. Berhanu D, Okwaraji YB, Belayneh AB, Lemango ET, Agonafer N, Birhanu BG, et al. Protocol for the evaluation of a complex intervention aiming at increased utilisation of primary child health services in Ethiopia: a before and after study in intervention and comparison areas. BMC Health Serv Res. 2020;20(1):339.
21. Central Statistical Agency (CSA) and ICF International. Ethiopian Demogrpahic and Health Survey 2011. Addis Ababa, Ethioia and Calvertn Marlyand, USA. CSA and ICF. 2012.
22. Armstrong Schellenberg JR, Adam T, Mshinda H, Masanja H, Kabadi G, Mukasa O, et al. Effectiveness and cost of facility-based Integrated Management of Childhood Illness (IMCI) in Tanzania. Lancet. 2004;364(9445):1583-94.
23. Berhanu D, Avan BI. Community Based Newborn Care: Baseline report summary, Ethiopia Octboer 2014. London: IDEAS London School of Hygiene & Tropical Medicine. 2014. . 2014.
24. Vyas S, Kumaranayake L. Constructing socio-economic status indices: how to use principal components analysis. Health Policy Plan. 2006;21(6):459-68.
25. Barry D, Frew AH, Mohammed H, Desta BF, Tadesse L, Aklilu Y, et al. The effect of community maternal and newborn health family meetings on type of birth attendant and completeness of maternal and newborn care received during birth and the early postnatal period in rural Ethiopia. J Midwifery Womens Health. 2014;59 Suppl 1:S44-54.
26. Tesfaye S, Barry D, Gobezayehu AG, Frew AH, Stover KE, Tessema H, et al. Improving coverage of postnatal care in rural Ethiopia using a community-based, collaborative quality improvement approach. J Midwifery Womens Health. 2014;59 Suppl 1:S55-64.
27. World Health Organization and International Initiative for Impact Evaluation. An evidence map of social, behavioural and community engagement interventions for reproductive, maternal, newborn and child health. Geneva: World Health Organization. 2017.
28. Rowe AK, Rowe SY, Peters DH, Holloway KA, Chalker J, Ross-Degnan D. Effectiveness of strategies to improve health-care provider practices in low-income and middle-income countries: a systematic review. Lancet Glob Health. 2018;6(11):e1163-e75.
29. Miller NP, Amouzou A, Hazel E, Legesse H, Degefie T, Tafesse M, et al. Assessment of the impact of quality improvement interventions on the quality of sick child care provided by Health Extension Workers in Ethiopia. J Glob Health. 2016;6(2):020404.
30. George A, Rodríguez DC, Rasanathan K, Brandes N, Bennett S. iCCM policy analysis: strategic contributions to understanding its character, design and scale up in sub-Saharan Africa. Health Policy and Planning. 2015;30(suppl_2):ii3-ii11.
31. Amouzou A, Kanyuka M, Hazel E, Heidkamp R, Marsh A, Mleme T, et al. Independent Evaluation of the integrated Community Case Management of Childhood Illness Strategy in Malawi Using a National Evaluation Platform Design. Am J Trop Med Hyg. 2016;94(3):574-83.
32. Campbell H, El Arifeen S, Hazir T, O'Kelly J, Bryce J, Rudan I, et al. Measuring coverage in MNCH: challenges in monitoring the proportion of young children with pneumonia who receive antibiotic treatment. PLoS Med. 2013;10(5):e1001421.
33. Hazir T, Begum K, El Arifeen S, Khan AM, Huque MH, Kazmi N, et al. Measuring coverage in MNCH: a prospective validation study in Pakistan and Bangladesh on measuring correct treatment of childhood pneumonia. PLoS Med. 2013;10(5):e1001422.

For peer review only - http://bmjopen.bmj.com/site/about/guidelines.xhtml

34. Persson LA, Nga NT, Malqvist M, Thi Phuong Hoa D, Eriksson L, Wallin L, et al. Effect of Facilitation of Local Maternal-and-Newborn Stakeholder Groups on Neonatal Mortality: Cluster-Randomized Controlled Trial. PLoS Med. 2013;10(5):e1001445.
35. Osrin D, Prost A. Perinatal interventions and survival in resource-poor settings: which work, which don't, which have the jury out? Arch Dis Child. 2010;95(12):1039-46.
36. Hawe P. Lessons from complex interventions to improve health. Annu Rev Public Health. 2015;36:307-23.
37. Johnson AD, Thiero O, Whidden C, Poudiougou B, Diakité D, Traoré F, et al. Proactive community case management and child survival in periurban Mali. BMJ Global Health. 2018;3(2):e000634.
38. Freeman PA, Schleiff M, Sacks E, Rassekh BM, Gupta S, Perry HB. Comprehensive review of the evidence regarding the effectiveness of community-based primary health care in improving maternal, neonatal and child health: 4. child health findings. J Glob Health. 2017;7(1):010904.
39. Ashebir F, Medhanyie AA, Mulugeta A, Persson LA, Berhanu D. Women's development group leaders' promotion of maternal, neonatal and child health care in Ethiopia: a cross-sectional study. Global Health Action. 2020;13(1):1748845.
40. Perry H, Morrow M, Davis T, Borger S, Weiss J, DeCoster M, et al. Care Groups II: A Summary of the Child Survival Outcomes Achieved Using Volunteer Community Health Workers in Resource-Constrained Settings. Glob Health Sci Pract. 2015;3(3):370-81.
41. Wuneh AD, Medhanyie AA, Bezabih AM, Persson LA, Schellenberg J, Okwaraji YB. Wealth-based equity in maternal, neonatal, and child health services utilization: a cross-sectional study from Ethiopia. Int J Equity Health. 2019;18(1):201.
42. Defar A, Okwaraji YB, Tigabu Z, Persson LA, Alemu K. Geographic differences in maternal and child health care utilization in four Ethiopian regions; a cross-sectional study. Int J Equity Health. 2019;18(1):173.

For peer review only

For peer review only - http://bmjopen.bmj.com/site/about/guidelines.xhtml

Table 1 Optimizing the Health Extension Program intervention implemented in 26 districts of Ethiopia, the assumptions and the expected intermediate indicators and outcomes.

Assumptions		
• Health managers and political leaders at all levels will be committed to supporting the intervention<br>• Strong coordination and partnership among the stakeholders at all levels<br>• Community influencers (religious and traditional leaders) will be change agents in promoting maternal, newborn and child health services<br>• Public sector and supply chain partners will ensure drug and service availability		
Intervention		
Community engagement	Primary care level capacity building	Ownership and accountability
• Health post open house<br>• Group discussions led by Women's Development Army leaders<br>• Engaging schools<br>• Engaging religious and traditional leaders<br>• Engaging agricultural extension workers, to reach male partners<br>• Educational health films<br>• Radio spots<br>• Printed information and education communication materials	**Women's Development Army:**<br>• Level one training<br>• Provision of job aids and tools<br>• Community based data for decision-making training for Women's Development Army leaders<br><br>**Health Extension Workers:**<br>• Gap-filling training and job aids<br>• Supportive supervision<br>• Performance review and clinical mentoring meetings<br>• Community based data for decision making training of trainers for Health Extension Workers	• Advocacy for the integration of Community-Based Newborn Care and integrated Community Case Management into planning, budgeting, management, and information systems<br>• District level annual based planning<br>• Management standards for health post opening hours<br>• Ambulance service for children's referral<br>• Engage kebele (sub-district) command post in the efforts<br>• Community forum<br>• Establish community feedback mechanisms
Intermediate indicators		
Caregivers knowledge, practices and community participation on matters relating to child health	Facility readiness in terms of medicine, equipment and supplies as well as health worker training and supervision necessary to provide child health services	Planning and monitoring of child health services in the district health system (availability of ambulance, Community Based Newborn Care and integrated Community Case Management indicators, and standardized operational hours for health posts).
Outcomes		
1. Primary outcome<br>Service utilization from an appropriate provider for sick children between the ages of 2-59 months<br>2. Secondary outcomes<br>Appropriate treatment for diarrhoea and pneumonia<br>Care-seeking for infants who were ill in the neonatal period<br>Appropriate treatment for possible serious bacterial infection in the neonatal period		

For peer review only - http://bmjopen.bmj.com/site/about/guidelines.xhtml

Table 2 Characteristics of mothers or caregivers of children aged 2-59 months and of their children at baseline (December 2016- February 2017) and endline surveys (December 2018-February 2019) in intervention and comparison areas.

	Baseline household survey				Endline household survey				
	Intervention		Comparison		Intervention		Comparison		
	N	% (95%CI)	N	% (95%CI)	N	% (95%CI)	N	% (95%CI)	P-value[a]
**Caregiver's characteristic**									
**Age (years)**	**1259**		**1273**		**2454**		**2275**		
<25		24(22, 27)		27(25, 30)		17(16, 19)		20(19, 22)	0.15[b]
25-29		28(26, 31)		30(27,33)		28(26, 29)		26(25, 28)	
30-34		19(17, 22)		17(15, 19)		23(21, 24)		21(19, 23)	
35-39		16(14, 19)		16(14, 18)		19(17, 21)		18(16, 19)	
>=40		12(10, 14)		10(8, 12)		13(12, 15)		14(13, 16)	
**Education**	**1259**		**1273**		**2454**		**2275**		
Schooling		36(34,39)		45(43, 48)		37(35,39)		45(42,47)	0.07[c]
No schooling		64(61, 66)		55(52, 57)		63(61, 65)		55(53, 58)	
**Religion**	**1259**		**1273**		**2454**		**2275**		
Orthodox Christians		54(51, 57)		38(36, 41)		61(59, 63)		47(45, 49)	0.04[c]
Others[d]		46(43, 49)		62(59, 64)		38(37, 41)		53(51, 55)	
**Socio-economic status**	**1259**		**1273**		**2454**		**2275**		
Q1(poorest)		19(17, 22)		23(21, 25)		18(17, 20)		25(23, 27)	0.81[b]
Q2		18(16, 21)		20(18, 22)		19(18, 20)		22(21, 25)	
Q3		20(18, 23)		18(16, 21)		19(18, 21)		18(16, 19)	
Q4		22(20, 25)		21(18, 23)		20(19, 22)		17(16, 19)	
Q5 (least poor)		19(18, 22)		18(16, 20)		23(22, 25)		17(16, 19)	
**Distance to the nearest HP**	**1104**		**1126**		**2030**		**1878**		<0.01[c]
<=30mins		59(56, 62)		71(69, 74)		64(62, 66)		64(62, 67)	
>30mins		40(39, 44)		29(26,31)		36(34, 38)		36(33,38)	
**Child 2-59 months of age**									
**Sex**	**1525**		**1471**		**2960**		**2740**		
Male		52(49,54)		51(48, 54)		52(50,53)		51(49,52)	0.77[c]
Female		48(46,51)		49(46,51)		48(47,50)		49(48,51)	
**Age (months)**	**1488**		**1453**		**2960**		**2740**		
2-11		19(17,21)		18(16, 20)		16(14,17)		15(14,16)	0.43[b]
12-23		19(17,21)		20(18, 22)		18(17,20)		16(15,18)	
24-35		23(21,25)		22(20, 24)		20(19,21)		19(18,20)	
36-47		24(21,26)		26(24, 28)		27(26,29)		27(26,29)	
48-59		16(14,18)		14(13, 16)		19(17,20)		22(21,24)	

[a] All models were adjusted for clustering

[b] P-value obtained from linear regression model for the variable to assess whether there was any difference between the groups that changed over time.

[c] P-value obtained from logistic regression model for the variable to assess whether there was any difference between the groups that changed over time.

[d] Includes Catholics, Muslims and Protestants.

For peer review only - http://bmjopen.bmj.com/site/about/guidelines.xhtml

Table 3 Characteristics of mothers and their children born in the 12 months prior to the survey at baseline (December 2016- February 2017) and endline surveys (December 2018-February 2019) in intervention and comparison areas.

	**Baseline household survey**				**Endline household survey**				
	Intervention		Comparison		Intervention		Comparison		
	N	% (95%CI)	N	% (95%CI)	N	% (95%CI)	N	% (95%CI)	P-value[a]
**Mother's characteristic**									
**Age (years)**	**331**		**331**		**482**		**400**		
<25		34(29, 40)		34(29, 39)		25(21, 29)		29(25, 34)	0.73[b]
25-29		37(3, 43)		38(32,43)		37(33, 42)		33(29, 38)	
30-34		16(13, 21)		15(12, 19)		22(19, 26)		22(18, 27)	
>=35		12(9, 16)		13(10, 17)		16(13, 20)		16(12, 20)	
**Education**	**331**		**331**		**482**		**400**		0.41[c]
Schooling		42(36, 47)		50(45, 56)		44(40, 49)		54(49, 59)	
No schooling		58(53, 64)		50(44, 55)		56(51, 60)		46(41, 51)	
**Religion**	**331**		**331**		**482**		**400**		
Orthodox Christians		47(41, 52)		32(27, 37)		57(52, 61)		41(36, 46)	0.22[c]
Others[d]		53(48, 52)		68(63, 73)		43(39, 48)		59(54, 64)	
**Socio-economic status**	**331**		**331**		**482**		**400**		
Q1(poorest)		21(17, 26)		21(17, 26)		21(17, 25)		29(25, 34)	0.29[b]
Q2		20(16, 25)		18(14, 23)		22(18,26)		2(18, 26)	
Q3		22(18, 27)		17(14, 22)		18(15, 22)		16(13, 20)	
Q4		17(13, 21)		21(16, 26)		19(15, 22)		19(11, 23)	
Q5(least poor)		20(16, 25)		22(18, 27)		21(17, 25)		14(13, 18)	
**Distance to the nearest HP**	**304**		**303**		**413**		**343**		0.16[c]
<=30mins		60(54, 65)		69(64, 74)		61(56, 66)		66(61, 71)	
>30mins		40(35, 46)		31(26, 36)		39(34, 44)		34(29, 39)	
**Child born in the last 12 months**									
**Sex**	**324**		**328**		**482**		**399**		
Male		49(43,54)		52(46, 57)		55(50,59)		52(47,57)	0.64[c]
Female		51(46,57)		48(43,54)		45(41, 50)		48(43,53)	
**Age (months)**	**331**		**331**		**482**		**400**		
0-2		21(17,26)		26(22,31)		22(18,25)		22(19,27)	0.02[b]
3-5		24(20,29)		32(27, 37)		25(21,29)		24(20,28)	
6-8		30(25,35)		23(18, 28)		26(23,30)		29(25,33)	
9-11		25(21,30)		19(15, 24)		27(23,31)		25 (21,29)	

[a] All models were adjusted for clustering
[b] P-value obtained from linear regression model for the variable to assess whether there was any difference between the groups that changed over time.
[c] P-value obtained from logistic regression model for the variable to assess whether there was any difference between the groups that changed over time.
[d] Includes Catholics, Muslims and Protestants.

For peer review only - http://bmjopen.bmj.com/site/about/guidelines.xhtml

Table 4 Child health training for health extension workers and Women's Development Army leaders at baseline (December 2016- February 2017) and endline surveys (December 2018-February 2019) in intervention and comparison areas

	**Baseline frontline worker survey**		**Endline frontline worker survey**			
	Intervention	Comparison	Intervention	Comparison	Difference in Difference[a]	P-value[b]
**Training**	% (95%CI) [n]	% (95%CI) [n]	% (95%CI) [n]	% (95%CI) [n]	%	
iCCM[c] for health extension workers	83 (74-90) [145]	82 (74-88) [131]	78 (69-85) [141]	80 (70-87) [133]	-3	0.63
CBNC[d] for health extension workers	64 (54-73) [145]	66 (56-74) [130]	69 (61-76) [141]	69 (60-77) [133]	2	0.89
MNCH[e] orientation for Women's Development Army leaders	70 (60-74) [93]	59 (48-68) [94]	62 (53-70) [169]	61 (52-70) [167]	-10	0.34

[a] Difference-in-Difference: the difference in the proportion between intervention and comparison areas at endline subtracted from the difference in proportion between intervention and comparison at baseline.

[b] P-value obtained from a logistic regression model for the difference-in-difference analysis

[c] iCCM: integrated community case management training

[d] CBNC: community-based newborn care management training

[e] MNCH: maternal, newborn, and child health orientation in the last 12 months

For peer review only - http://bmjopen.bmj.com/site/about/guidelines.xhtml

Table 5: Observed availability of infrastructure, equipment, supplies and drugs for treatment of childhood illness at health posts and health centres at baseline (December 2016- February 2017) and endline surveys (December 2018-February 2019) in intervention and comparison areas

	Health centre						Health post					
	Baseline		Endline			P-value[b]	Baseline		Endline			P-value[b]
	Intervention n=74	Comparison n=81	Intervention n=68	Comparison n=74	Difference in Difference [a]		Intervention n=84	Comparison n=85	Intervention n=79	Comparison n=86	Difference in Difference [a]	
	% (95%CI)	% (95%CI)	% (95%CI)	% (95%CI)	%		% (95%CI)	% (95%CI)	% (95%CI)	% (95%CI)	%	
**Infrastructure**												
Patient toilet	99 (91-100)	96 (89-99)	97 (89-99)	97 (90-99)	-3	0.51	87 (78-93)	80(70-87)	82(72-89)	81 (72-88)	-6	0.54
Water	91 (82-96)	74 (63-82)	75 (63-84)	69 (57-79)	-11	0.36	69 (58-78)	55(44-66)	51(40-62)	52 (42-63)	-15	0.43
Fridge	77(66-85)	78(67-86)	78(66-86)	80(69-86)	-1	0.838	10(5-18)	9(10-18)	33(23-44)	40(30-50)	-8	0.027
Sterilizer	70(57-80)	63(52-73)	74(62-83)	68(59-77)	-1	0.751	10(5-18)	7(3-15)	10(5-19)	10(5-19)	-3	0.515
Electricity	59(48-70)	62(51-72)	65(52-75)	70(57-79)	-2	0.588	17(10-26)	19(12-29)	23(15-34)	25(17-36)	0	0.392
**Drugs**												
Amoxicillin [c]	97(90-99)	99(91-100)	97(89-99)	99(81-100)	0	0.975	79(68-86)	81(71-88)	73(62-82)	80(70-87)	-5	0.85
Co-trimoxazole	97 (90-99)	98 (90-99)	94 (85-98)	95 (86-98)	0	0.67	40 (30-51)	15 (9-25)	8 (3-16)	6 (2-13)	-23	0.97
Gentamicin [d]	82 (72-90)	81 (71-89)	94 (85-98)	97 (90-99)	-4	0.07	38 (28-49)	41 (31-52)	37 (27-48)	15 (9-25)	25	<0.01
Ampicillin[e]	84 (73-91)	90 (81-95)	97 (89-99)	99 (91-100)	4	0.31						
ORS [f]	89 (80-95)	80 (70-88)	93 (83-97)	92 (83-97)	-8	0.16	76 (66-84)	58 (47-68)	92 (84-97)	85 (75-91)	-11	0.22
Zinc	12 (6-22)	7 (3-16)	16 (9-287	16 (9-27)	-5	0.21	82 (72-88)	80 (70-87)	84 (73-90)	80 (70-87)	2	0.93
Zinc-ORS[e] combined	74 (63-83)	77 (66-85)	88 (78-94)	68 (56-74)	23	0.03	10 (5-18)	5 (2-12)	14 (08-24)	9 (4-16)	0	0.59
Malaria RDT [g]	88 (78-94)	77 (66-85)	87 (76-93)	65 (53-75)	11	0.23	75 (64-83)	48 (37-59)	65 (53-74)	37 (27-48)	1	0.56
RUTF [h]	82 (72-90)	100	88 (78-94)	99 (91-100)	7		5 (2-12)	11 (06-19)	47 (36-58)	74 (64-83)	-21	<0.01
**Equipment**												
Stethoscope	100	100	99 (90-100)	100	-1		74 (63-82)	81 (71-88)	72 (61-81)	56 (45-66)	23	<0.01
MUAC [i] tape	100	99 (91-100)	99 (90-100)	99 (91-100)	-1		98 (91-99)	100	97 (90-99)	100	-1	0.95
Thermometer	100	98 (90-99)	100	99 (91-100)	-1		86 (76-92)	87 (78-93)	85 (75-91)	76 (65-84)	10	0.11
Infant scale	96 (88-99)	96 (89-99)	97 (89-99)	99 (91-100)	-2	0.49	80 (70-87)	81 (71-88)	80 (69-87)	77 (66-85)	4	0.52
Ambu face mask	95 (86-98)	94 (86-97)	96 (87-99)	96 (88-99)	-1	0.69	81 (71-88)	71 (60-79)	23 (15-34)	13 (7-22)	0	
Weighing sling	86 (76-93)	95 (87-98)	93 (83-97)	92 (83-97)	10	0.23	70 (59-79)	76 (66-84)	75 (64-83)	78 (68-86)	3	0.94
Syringes and needle	100	95 (87-98)	100	100	-5		85 (75-91)	81 (71-88)	78 (68-86)	73 (63-82)	1	0.55
Functional timer [j]							21 (14-32)	22 (15-33)	5 (2-13)	16 (10-26)	-10	0.38
**Job aids**												
0-2 months registration book	95 (86-98)	93 (84-97)	97 (89-99)	95 (86-98)	0	0.98	92 (83-96)	88 (79-94)	94 (85-97)	86 (77-92)	4	0.53

For peer review only - http://bmjopen.bmj.com/site/about/guidelines.xhtml

2-59 months registration book	100	99 (91-100)	96 (87-99)	100	-5		95 (88-98)	89 (81-94)	95 (87-98)	94 (87-98)	-5	0.31
Chart booklet	96 (88-99)	95 (87-98)	99 (89-100)	95 (86-98)	3	0.50	87 (78-93)	87 (78-93)	94 (85-97)	90 (81-95)	4	0.77
Supervision checklist [k]	92 (83-96)	93 (84-97)	90 (80-95)	85 (75-92)	6	0.28						
Family health card [l]							83 (74-90)	87 (78-93)	81 (71-88)	81 (72-88)	4	0.45

[a] Difference-in-Difference: the difference in the proportion between intervention and comparison areas at endline subtracted from the difference in proportion between intervention and comparison at baseline.

[b] P-value obtained from a logistic regression model for the difference-in-difference analysis

[c] Amoxicillin 250mg, 125 mg, or 125 mg suspension

[d] Gentamicin 80mg/2ml or 20mg/2ml for the health centre and 20mg/2ml for health posts

[e] Health posts are not expected to have stocks of ampicillin and was hence not assessed

[f] ORS: oral rehydration solution

[g] RDT: rapid diagnostic test

[h] RUTF: ready-to-use therapeutic food

[i] MUAC: middle upper arm circumference

[j] Functional timer not assessed at health centre

[k] Supervision checklist was not assessed at health posts as stocks are expected to be kept at health centres

[l] Family health card was not assessed at health centres as it is a tool mainly used by health extension workers

Table 6 Median number of newborns (0-2 months) and children (2-59 months) registered in health centres and health posts in the quarter prior to survey at baseline (December 2016- February 2017) and endline surveys (December 2018-February 2019) in intervention and comparison areas

	**Health centre**						**Health post**					
	**Baseline facility survey**		**Endline facility survey**		Difference in Difference*	P-value	**Baseline facility survey**		**Endline facility survey**		Difference in Difference*	P-value[a]
	Intervention n=67	Comparison n=88	Intervention n=61	Comparison n=81			Intervention n=74	Comparison n=95	Intervention n=69	Comparison n=96		
**Age group**	Median (IQR)	Median (IQR)	Median (IQR)	Median (IQR)	Median		Median (IQR)	Median (IQR)	Median (IQR)	Median (IQR)	Median	
0-59 days	5 (8)	3(6)	9 (11)	3 (6)	4	0.08	0 (2)	0 (1)	0 (3)	0 (0)	0	>0.99
2-59 months	237 (309)	149 (171)	232 (340)	128 (119)	16	0.58	18 (37)	13 (25)	22 (33)	10 (24)	7	0.22

[a] Difference-in-Difference: the difference in the median children seen in the three months prior to the survey between intervention and comparison area health facilities at endline subtracted from the difference in the median between intervention and comparison area health facilities at baseline.

[b] P-value obtained from using quantile regression analysis.

For peer review only - http://bmjopen.bmj.com/site/about/guidelines.xhtml

Table 7 Illness, care-seeking and case-management in the two weeks prior to the survey among children 2-59 months of age reported by caregivers from the household survey at baseline (December 2016- February 2017) and endline surveys (December 2018-February 2019) in intervention and comparison areas.

	Baseline household survey		Endline household survey		Difference in Difference[a]	OR (95% CI)	Adjusted OR (95% CI) [b]
	Intervention	Comparison	Intervention	Comparison			
	% (95%CI) [n]	% (95%CI) [n]	% (95%CI) [n]	% (95%CI) [n]	%		
Illness in the last 2 weeks	6 (5-8) [n=1434]	5 (4-8) [n=1440]	13 (11-15) [n=2932]	11(9-14) [n=2707]	n/a [c]	n/a [c]	n/a [c]
Among ill							
Care-seeking for any illness[d]	58 (47-68) [n=88]	49 (39-60) [n=79]	39 (32-45) [n=381]	34 (27-41) [n=308]	-4	0.62 (0.19-2.04)	0.49 (0.12-1.95)
ORS with zinc for reported diarrhoea	32 (14-56) [n=19]	25 (10-50) [n=20]	38 (25-51) [n=72]	30 (19-42) [n=47]	1	0.74 (0.04-12.26)	0.70 (0.04-13.66)
ORS with or without zinc for reported diarrhoea	53 (30-74) [n=19]	40 (18-66) [n=20]	50 (38-62) [n=72]	53 (38-68) [n=47]	-16	0.32 (0.02-4.90)	0.29 (0.02-5.33)
Antibiotic for possible pneumonia [e] symptoms	67 (16-95) [n=9]	56 (22-85) [n=9]	62 (38-81) [n=21]	69 (42-87) [n=19]	-18	0.29 (0.00-26.83)	0.15 (0.00-16.18)

[a] Difference-in-Difference: the difference in the proportion between intervention and comparison areas at endline subtracted from the difference in proportion between intervention and comparison at baseline.

[b] Adjusted for religion and distance to nearest health post. Sample size is smaller for adjusted ORs: care-seeking =127 missing data on distance; diarrhoea treatment =23 missing data on distance; antibiotics for possible pneumonia =6 missing data on distance

[c] The difference-in-difference, OR and adjusted OR were not calculated for any illness in the last two weeks as this was not a an outcome assumed to be influenced by the intervention

[d] Care-seeking from hospital, health centre, health post or private clinic

[e] Possible pneumonia cases are those children with reported cough and fast breathing or difficult breathing, but not due to a blocked nose.

For peer review only - http://bmjopen.bmj.com/site/about/guidelines.xhtml

Table 8 Morbidity, care-seeking and management among infants in the neonatal period at baseline (December 2016- February 2017) and endline surveys (December 2018-February 2019) in intervention and comparison areas.

	**Baseline household survey**		**Endline household survey**		Difference in Difference[a]	OR (95% CI)	Adjusted OR (95% CI) [b]
	Intervention	Comparison	Intervention	Comparison			
	% (95%CI) [n]	% (95%CI) [n]	% (95%CI) [n]	% (95%CI) [n]	%		
Among all							
Baby ill in the first month of life	14 (11-19) [n=331]	5 (3 -9) [n=331]	9 (7-13) [n=482]	8 (6-11) [n=397]	n/a [c]	n/a [c]	n/a [c]
**Among ill**							
Prevalence of possible neonatal sepsis [e]	83 (70-91) [n=47]	78 (48-93) [n=18]	73 (59-84) [n=45]	84 (65-94) [n=32]	n/a [d]	n/a [d]	n/a [d]
Care-seeking for ill baby [f]	74 (59-86) [n=47]	44 (23-69) [n=18]	51 [g] (34-68) [n=43]	68 [h] (49-82) [n=31]	-47	0.04 (0.00-0.49)	0.04 (0.00-0.60)
**Among possible sepsis**							
Any antibiotic	51 (34-68) [n=39]	36 (17-60) [n=14]	57 (38-73) [n=30]	68 (43-85) [n=25]	-26	0.17 (0.01-2.06)	0.19 (0.02-2.42)

[a] Difference-in-Difference: the difference in the proportion between intervention and comparison areas at endline subtracted from the difference in proportion between intervention and comparison at baseline.
[b] Adjusted for age of infant
[c] The difference-in-difference, OR and adjusted OR were not calculated for baby ill in the first month of life as this was not a an outcome assumed to be influenced by the intervention
[d] The difference-in-difference, OR and adjusted OR were not calculated for prevalence of possible neonatal sepsis as this was not a an outcome assumed to be influenced by the intervention
[e] Possible neonatal sepsis: fever, unable to suckle/feed difficult/fast breathing, severe chest in-drawing, convulsions or lethargy
[f] Care-seeking outside the home
[g] Missing data for two individuals
[h] Missing data for one individual

For peer review only

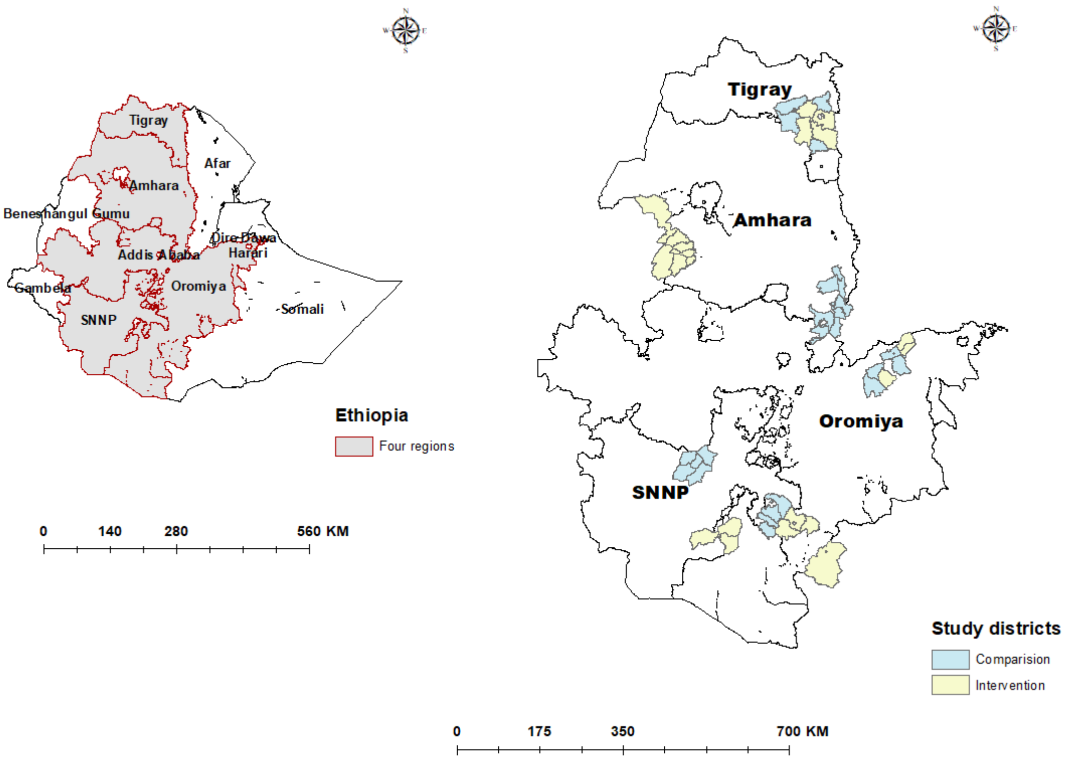

For peer review only

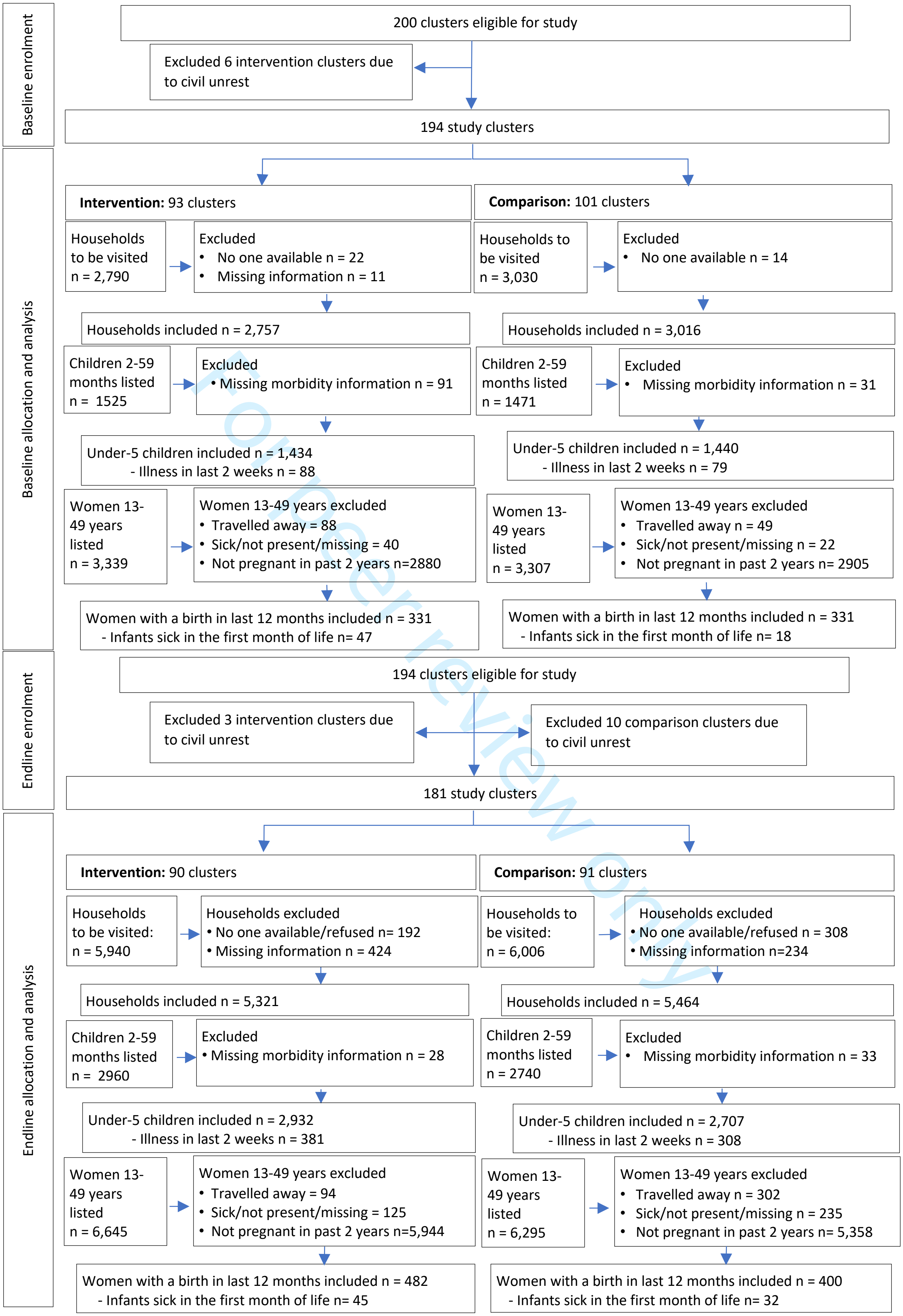

For peer review only

S1 Table Demographic characteristics, number of different health facilities and staff at baseline (December 2016-February 2017) and endline (December 2018-February 2019) surveys in intervention and comparison districts.

**Characteristic**	**Baseline**		**Endline**	
	Intervention districts n=21[a]	Comparison districts n=25[b]	Intervention districts n=22[c]	Comparison districts n=24 [d]
	Mean (95% CI)	Mean (95% CI)	Mean (95% CI)	Mean (95% CI)
Population size (*1000)	132 (111-152)	139 (121-159)	121 (103-141)	138 (115-162)
Women 15-49 years (*1000)	29 (25-34)	32 (27-38)	28 (22-34)	31 (25-36)
Children <5 years (*1000)	20 (17-23)	21 (18-25)	19 (16-23)	21 (16-26)
Household size	4.8 (4.6-5.0)	4.6 (4.5-4.7)	4.5 (4.2-4.8)	4.6 (4.4-4.9)
Hospitals	0.4 (0.2-0.6)	0.3 (0.1-0.5)	0.3 (0.1-0.5)	0.4 (0.2-0.6)
Health centres	5.3 (4.8-5.8)	5.0 (4.6-5.5)	5.0 (4.4-5.6)	4.8 (4.1-5.5)
Health posts	24 (21-27)	27 (24-30)	22 (19-26)	26 (22-30)
Ambulances	1.6 (1.3-2.0)	1.9 (1.6-2.3)	2.4 (2.1-2.8)	3.1 (2.4-3.8)
Ambulances for child use	1.3 (0.8-1.7)	1.5 (1.0-2.0)	2.0 (1.5-2.5)	2.8 (2.0-3.7)
Health officers per health centre	2.0 (1.7-2.3)	1.9 (1.6-2.3)	2.6 (2.0-3.1)	2.4 (1.9-2.8)
Midwives per health centre	2.5 (2.1-3.0)	2.5 (2.0-3.2)	3.7 (2.6-4.7)	3.1 (2.4-3.8)
Health extension workers per health post	2.3 (2.0-2.7)	2.2 (2.0-2.3)	2.6 (2.3-2.9)	2.8 (1.4-4.2)

[a] Missing data from 5 districts
[b] Missing data from 1 district
[c] Missing data from 4 districts
[d] Missing data from 2 districts

For peer review only

S2 Table Health messages received, meetings attended, and health issues addressed in meetings among households participating at baseline (December 2016- February 2017) and endline surveys (December 2018-February 2019) in intervention and comparison areas

Categories	Baseline household survey		Endline household survey			
	Intervention N=1240	Comparison N=1264	Intervention N=2434	Comparison N=2247	Difference in Difference[a]	P-value[b]
**Seen or heard messages on**						
Diarrhoea treatment	60 (55-65)	49 (43-55)	52 (49-56)	44 (40-48)	-3	0.03
Pneumonia treatment	29 (24-35)	27 (22-32)	26 (22-30)	22 (18-26)	2	0.76
Sick newborn treatment	39 (33-46)	34[c] (28-40)	31 (26-35)	27 (23-31)	-1	0.87
**Participated in developing local maternal, newborn and child health action plan in the last 12 months organized by[c]:**						
Kebele health team	1 (1-2)	1 (0-1)	1 (0-2)	1 (0-2)	0	0.25
Health extension worker	3 (2-5)	1 (1-2)	1 (1-2)	1 (0-1)	-2	0.04
**Attended meeting to discuss on maternal, newborn and child health issues in the past 12 months organized by[d]:**						
Kebele health team	2 (1-3)	1 (0-2)	1 (1-2)	1 (0-1)	-1	0.08
Health extension worker	3 (2-5)	2 (1-3)	2 (1-2)	1 (0-1)	0	0.31

[a] Difference-in-Difference: the difference in the proportion between intervention and comparison areas at endline subtracted from the difference in proportion between intervention and comparison at baseline.

[b] P-value obtained from a logistic regression model for the Difference-in-Difference analysis

[c] Missing data from one individual

[d] Missing data for one individual in intervention areas and 11 individuals in comparison areas at baseline and for one individual in intervention and comparison areas each at endline.

[e] Missing data for two individuals in intervention areas and 11 individuals in comparison areas at baseline and for one individual in intervention and comparison areas each at endline.

For peer review only

S3 Table Caregivers' unprompted knowledge of newborn danger signs at baseline (December 2016-February 2017) and endline surveys (December 2018-February 2019) in intervention and comparison areas.

	Baseline household survey		Endline household survey		Difference in Difference[a]	P-value[b]
	Intervention n=1259	Comparison n=1273	Intervention n=2434	Comparison n=2247		
	% (95%CI)	% (95%CI)	% (95%CI)	% (95%CI)	%	
**Signs of newborn illness**						
Reduced feeding	60 (55-65)	59 (53-64)	53 (49-58)	51 (47-56)	1	0.16
Difficult or fast breathing	31 (26-36)	38 (33-43)	37[c] (34-41)	35 (32-38)	9	0.06
Movement only when stimulated or no moment even when stimulated	11 (8-15)	20 (16-24)	18[c] (16-21)	19 (16-23)	8	<0.01
Unusually hot or cold	49 (43-54)	42 (38-48)	64[c] (60-69)	59 (53-64)	-2	0.05
Convulsions	7 (5-9)	9 (6-12)	14[c] (12-17)	12 (10-15)	4	0.35
Severe chest in-drawing	6 (4-8)	4 (2-6)	10[c] (8-12)	7 (5-10)	1	0.33
Yellow palms/soles/eyes	1 (1-2)	2 (1-4)	7[c] (5-9)	6 (3-8)	2	0.30
Diarrhoea	54 (50-59)	52 (47-58)	60[c] (55-64)	53 (49-57)	5	0.59
Skin pustules	5 (3-7)	7 (5-9)	10[c] (8-13)	8 (6-11)	4	0.03
Cord red or draining pus	1 (1-2)	1 (1-2)	6[c] (4-8)	5 (3-7)	1	0.56
Small infant (weigh less than 2000 grams)	1 (1-2)	1 (0-1)	5[c] (3-7)	4 (2-6)	1	0.83

[a] Difference-in-Difference: the difference in the proportion between intervention and comparison areas at endline subtracted from the difference in proportion between intervention and comparison at baseline.
[b] P-value obtained from a logistic regression model for the Difference-in-Difference analysis
[c] Missing data from one individual

For peer review only

S4 Table Caregivers' knowledge on actions to be taken when a child under five years is sick at baseline (December 2016- February 2017) and endline surveys (December 2018-February 2019) in intervention and comparison areas.

	**Baseline household survey**		**Endline household survey**			
	Intervention n=1259	Comparison n=1273	Intervention n=2434	Comparison n=2247	Difference in Difference[a]	P-value[b]
	% (95%CI)	% (95%CI)	% (95%CI)	% (95%CI)	%	
**Action taken when child has:**						
**Fever**						
Go to health extension worker	20 (16-25)	22 (17-27)	26 (22-31)	25 (21-30)	3	0.26
Go to health centre	83 (79-87)	84 (79-87)	88 (85-90)	90 (87-92)	-1	0.77
**Diarrhoea**						
Go to health extension worker	20 (16-25)	21 (17-27)	27 (22-32)	24 (20-29)	4	0.48
Go to health centre	86 (82-89)	86 (81-89)	90 (86-92)	90 (88-92)	0	0.84
**Respiratory infection or cough**						
Go to health extension worker	20 (16-24)	22 (17-27)	26 (21-30)	24 (20-29)	4	0.64
Go to health centre	86 (83-89)	86 (82-90)	87 (83-89)	89 (86-91)	-2	0.62
**Signs of newborn sepsis[c]**						
Go to health extension worker	18 (14-22)	20 (15-26)	23 (19-27)	22 (18-26)	3	0.33
Go to health centre	87 (83-90)	86 (82-90)	87[d] (85-88)	89 (87-91)	-3	0.30

[a] Difference-in-Difference: the difference in the proportion between intervention and comparison areas at endline subtracted from the difference in proportion between intervention and comparison at baseline.
[b] P-value obtained from a logistic regression model for the Difference-in-Difference analysis
[c] Caregivers were read the dangers signs indicating neonatal sepsis: reduced feeding, difficult or fast breathing, movement when stimulated or no movement even when stimulated, unusually hot or cold, convulsion, severe chest in-drawing
[d] Missing data from one individual

For peer review only

S5 Table Health post characteristics in baseline (December 2016- February 2017) and endline surveys (December 2018-February 2019) at intervention and comparison areas.

	Baseline health post survey		Endline health post survey			
	Intervention n=145	Comparison n=131	Intervention n=141	Comparison n=133	Difference in Difference[a]	P-value[b]
**Characteristics of health posts:**	% (95%CI)	% (95%CI)	% (95%CI)	% (95%CI)	%	
Fully staffed[c]	89 (82-93)	79 (70-85)	89 (82-93)	86 (78-91)	-7	0.55
Open less than five days a week	20 (13-29)	31 (21-42)	10 (6-17)	20 (13-30)	1	0.99
Operational days posted	34 (25-452)	31 (22-42)	23 (16-34)	16 (9-25)	4	0.76
Operational hours posted	27 (18-38)	25 (17-35)	13 (8-22)	13 (7-22)	-2	0.88

[a] Difference in Difference: the difference in the proportion between intervention and comparison areas at endline subtracted from the difference in proportion between intervention and comparison at baseline.
[b] P-value obtained from a logistic regression model for the Difference-in-Difference analysis
[c] Two or more health extension workers per health post

S6 Table Supervision at baseline (December 2016- February 2017) and endline surveys (December 2018-February 2019) in intervention and comparison areas.

	Baseline frontline worker survey		Endline frontline worker survey			
	Intervention	Comparison	Intervention	Comparison	Difference in Difference[a]	P-value[b]
**Supervision**	% (95%CI) [n]	% (95%CI) [n]	% (95%CI) [n]	% (95%CI) [n]	%	
**Health centre**: supervision received in the last 3 months	66 (55-76) [n=83]	61 (50-70) [n=92]	64 (52-75) [n=70]	62 (50-73) [n=71]	-3	0.72
**Health extension worker supervision:** supervision received in last month	59 (48-68) [n=145]	60 (49-69) [n=131]	57 (47-67) [n=141]	57 (47-67) [n=133]	1	0.90
**Health extension worker:** performance review and clincial mentoring meeting in the last 6 months	46 (36-55) [n=145]	41 (32-51) [n=131]	51 (42-60) [n=141]	41 (31-50) [n=133]	5	0.46
**Women's development army leaders:** met with health extension workers and other women development army leaders in the last 3 months	67 (56-76) [n=93]	60 (49-69) [n=94]	64 (55-72) [n=169]	60 (52-69) [n=167]	-3	0.357

[a] Difference-in-Difference: the difference in the proportion between intervention and comparison areas at endline subtracted from the difference in proportion between intervention and comparison at baseline.
[b] P-value obtained from a logistic regression model for the Difference-in-Difference analysis

For peer review only - http://bmjopen.bmj.com/site/about/guidelines.xhtml

CONSORT 2010 checklist of information to include when reporting a randomised trial*

Section/Topic	Item No	Checklist item	Reported on page No
**Title and abstract**			
	1a	Identification as a randomised trial in the title	Not randomized
	1b	Structured summary of trial design, methods, results, and conclusions (for specific guidance see CONSORT for abstracts)	1
**Introduction**			
Background and objectives	2a	Scientific background and explanation of rationale	6-7
	2b	Specific objectives or hypotheses	7
**Methods**			
Trial design	3a	Description of trial design (such as parallel, factorial) including allocation ratio	8
	3b	Important changes to methods after trial commencement (such as eligibility criteria), with reasons	N/A
Participants	4a	Eligibility criteria for participants	9
	4b	Settings and locations where the data were collected	7-8
Interventions	5	The interventions for each group with sufficient details to allow replication, including how and when they were actually administered	7 & Table 1 (page 28)
Outcomes	6a	Completely defined pre-specified primary and secondary outcome measures, including how and when they were assessed	11
	6b	Any changes to trial outcomes after the trial commenced, with reasons	NA
Sample size	7a	How sample size was determined	9
	7b	When applicable, explanation of any interim analyses and stopping guidelines	N/A
Randomisation:			
Sequence generation	8a	Method used to generate the random allocation sequence	N/A
	8b	Type of randomisation; details of any restriction (such as blocking and block size)	N/A
Allocation concealment mechanism	9	Mechanism used to implement the random allocation sequence (such as sequentially numbered containers), describing any steps taken to conceal the sequence until interventions were assigned	N/A
Implementation	10	Who generated the random allocation sequence, who enrolled participants, and who assigned participants to interventions	7
Blinding	11a	If done, who was blinded after assignment to interventions (for example, participants, care providers, those	10 & 13

For peer review only - http://bmjopen.bmj.com/site/about/guidelines.xhtml

		assessing outcomes) and how	
	11b	If relevant, description of the similarity of interventions	N/A
Statistical methods	12a	Statistical methods used to compare groups for primary and secondary outcomes	13
	12b	Methods for additional analyses, such as subgroup analyses and adjusted analyses	13
**Results**			
Participant flow (a diagram is strongly recommended)	13a	For each group, the numbers of participants who were randomly assigned, received intended treatment, and were analysed for the primary outcome	37 Figure 1
	13b	For each group, losses and exclusions after randomisation, together with reasons	37 Figure 1
Recruitment	14a	Dates defining the periods of recruitment and follow-up	8
	14b	Why the trial ended or was stopped	8
Baseline data	15	A table showing baseline demographic and clinical characteristics for each group	29 & 30
Numbers analysed	16	For each group, number of participants (denominator) included in each analysis and whether the analysis was by original assigned groups	29 & 30
Outcomes and estimation	17a	For each primary and secondary outcome, results for each group, and the estimated effect size and its precision (such as 95% confidence interval)	31-35
	17b	For binary outcomes, presentation of both absolute and relative effect sizes is recommended	34-35
Ancillary analyses	18	Results of any other analyses performed, including subgroup analyses and adjusted analyses, distinguishing pre-specified from exploratory	31-35
Harms	19	All important harms or unintended effects in each group (for specific guidance see CONSORT for harms)	N/A
**Discussion**			
Limitations	20	Trial limitations, addressing sources of potential bias, imprecision, and, if relevant, multiplicity of analyses	19
Generalisability	21	Generalisability (external validity, applicability) of the trial findings	19
Interpretation	22	Interpretation consistent with results, balancing benefits and harms, and considering other relevant evidence	19-22
**Other information**			
Registration	23	Registration number and name of trial registry	4
Protocol	24	Where the full trial protocol can be accessed, if available	8
Funding	25	Sources of funding and other support (such as supply of drugs), role of funders	24

*We strongly recommend reading this statement in conjunction with the CONSORT 2010 Explanation and Elaboration for important clarifications on all the items. If relevant, we also recommend reading CONSORT extensions for cluster randomised trials, non-inferiority and equivalence trials, non-pharmacological treatments, herbal interventions, and pragmatic trials. Additional extensions are forthcoming: for those and for up to date references relevant to this checklist, see www.consort-statement.org.

For peer review only - http://bmjopen.bmj.com/site/about/guidelines.xhtml
